# Peatland *Acidobacteria* with a dissimilatory sulfur metabolism

**DOI:** 10.1038/s41396-018-0077-1

**Published:** 2018-02-23

**Authors:** Bela Hausmann, Claus Pelikan, Craig W. Herbold, Stephan Köstlbacher, Mads Albertsen, Stephanie A. Eichorst, Tijana Glavina del Rio, Martin Huemer, Per H. Nielsen, Thomas Rattei, Ulrich Stingl, Susannah G. Tringe, Daniela Trojan, Cecilia Wentrup, Dagmar Woebken, Michael Pester, Alexander Loy

**Affiliations:** 1 Division of Microbial Ecology, Department of Microbiology and Ecosystem Science, Research Network Chemistry meets Microbiology, University of Vienna, Vienna, Austria; 2 Department of Biology, University of Konstanz, Konstanz, Germany; 3 Department of Chemistry and Bioscience, Center for Microbial Communities, Aalborg University, Aalborg, Denmark; 4 US Department of Energy Joint Genome Institute, Walnut Creek, CA USA; 5 Division of Computational Systems Biology, Department of Microbiology and Ecosystem Science, Research Network Chemistry meets Microbiology, University of Vienna, Vienna, Austria; 6 Department for Microbiology and Cell Science, Fort Lauderdale Research and Education Center, UF/IFAS, University of Florida, Davie, FL USA; 7 Leibniz Institute DSMZ, Braunschweig, Germany

## Abstract

Sulfur-cycling microorganisms impact organic matter decomposition in wetlands and consequently greenhouse gas emissions from these globally relevant environments. However, their identities and physiological properties are largely unknown. By applying a functional metagenomics approach to an acidic peatland, we recovered draft genomes of seven novel *Acidobacteria* species with the potential for dissimilatory sulfite (*dsrAB*, *dsrC*, *dsrD*, *dsrN*, *dsrT*, *dsrMKJOP*) or sulfate respiration (*sat*, *aprBA*, *qmoABC* plus *dsr* genes). Surprisingly, the genomes also encoded DsrL, which so far was only found in sulfur-oxidizing microorganisms. Metatranscriptome analysis demonstrated expression of acidobacterial sulfur-metabolism genes in native peat soil and their upregulation in diverse anoxic microcosms. This indicated an active sulfate respiration pathway, which, however, might also operate in reverse for dissimilatory sulfur oxidation or disproportionation as proposed for the sulfur-oxidizing *Desulfurivibrio alkaliphilus*. *Acidobacteria* that only harbored genes for sulfite reduction additionally encoded enzymes that liberate sulfite from organosulfonates, which suggested organic sulfur compounds as complementary energy sources. Further metabolic potentials included polysaccharide hydrolysis and sugar utilization, aerobic respiration, several fermentative capabilities, and hydrogen oxidation. Our findings extend both, the known physiological and genetic properties of *Acidobacteria* and the known taxonomic diversity of microorganisms with a DsrAB-based sulfur metabolism, and highlight new fundamental niches for facultative anaerobic *Acidobacteria* in wetlands based on exploitation of inorganic and organic sulfur molecules for energy conservation.

## Introduction

Specialized microorganisms oxidize, reduce, or disproportionate sulfur compounds of various oxidation states (–II to +VI) to generate energy for cellular activity and growth and thereby drive the global sulfur cycle. The capability for characteristic sulfur redox reactions such as dissimilatory sulfate reduction or sulfide oxidation is not confined to single taxa but distributed across different, often unrelated taxa. The true extent of the taxon-diversity within the different guilds of sulfur microorganisms is unknown [1]. However, ecological studies employing specific sulfur metabolism genes (e.g., dissimilatory adenylyl-sulfate reductase-encoding *aprBA*, dissimilatory sulfite reductase-encoding *dsrAB*, or *soxB* that codes for a part of the thiosulfate-oxidizing Sox enzyme machinery) as phylogenetic and functional markers have repeatedly demonstrated that only a minor fraction of the sulfur metabolism gene diversity in many environments can be linked to recognized taxa [2–4]. A systematic review of *dsrAB* diversity has revealed that the reductive bacterial-type enzyme branch of the DsrAB tree contains at least thirteen family-level lineages without any cultivated representatives. This indicates that major taxa of sulfate-/sulfite-reducing microorganisms have not yet been identified [3].

Wetlands are among those ecosystems that harbor a diverse community of microorganisms with reductive-type DsrAB, most of which cannot be identified because they are distant from taxonomically classified DsrAB sequences [5]. Sulfur-cycling microorganisms provide significant ecosystem services in natural and anthropogenic wetlands, which are major sources of the climate-warming greenhouse gas methane [6, 7]. While inorganic sulfur compounds are often detected only at low concentration (lower µM range), fast sulfur cycling nevertheless ensures that oxidized sulfur compounds such as sulfate are rapidly replenished for anaerobic respiration. The activity of sulfate-reducing microorganisms (SRM) fluctuates with time and space, but at peak times can contribute considerably to the anaerobic mineralization of organic carbon in wetlands [5]. Simultaneously, SRM prevent methane production by rerouting carbon flow away from methanogenic archaea. Peat microorganisms that are affiliated to known SRM taxa, such as *Desulfosporosinus*, *Desulfomonile*, and *Syntrophobacter*, are typically found in low abundance [8–15]. In contrast, some microorganisms that belong to novel, environmental dsrAB lineages can be considerably more abundant in wetlands than species-level *dsrAB* operational taxonomic units of known taxa [13]. However, the taxonomic identity of these novel *dsrAB*-containing microorganisms and their role in sulfur and carbon cycling has yet to be revealed.

To identify these unknown DsrAB-encoding organisms and further investigate their fundamental ecological niches, we recovered thirteen metagenome-assembled genomes (MAGs) encoding DsrAB from a peat soil through a targeted, functional metagenomics approach. We analyzed expression of predicted physiological capabilities of the MAGs by metatranscriptome analyses of anoxic peat soil microcosms that were periodically stimulated by small additions of individual fermentation products with or without supplemented sulfate [9]. Here, we show that facultatively anaerobic members of the diverse *Acidobacteria* community in wetlands employ one or more types of dissimilatory sulfur metabolism.

## Materials and methods

### Anoxic microcosm experiments, stable isotope probing, and nucleic acids isolation

DNA and RNA samples were retrieved from a previous peat soil microcosm experiment [9]. Briefly, soil from 10–20 cm depth was sampled from an acidic peatland (Schlöppnerbrunnen II, Germany) in September 2010, and stored at 4 °C for one week prior to nucleic acids extractions and set-up of soil slurry incubations. Individual soil slurry microcosms were incubated anoxically (100% N_2_ atmosphere) in the dark at 14 °C, and regularly amended with low amounts (<200 µM) of either formate, acetate, propionate, lactate, butyrate, or without any additional carbon sources (six replicates each). In addition, half of the microcosms for each substrate were periodically supplemented with low amounts of sulfate (initial amendment of 190–387 µM with periodic additions of 79–161 µM final concentrations). DNA and RNA were extracted from the native soil and RNA was additionally extracted from the soil slurries after 8 and 36 days of incubation.

Furthermore, DNA was obtained from the heavy, ¹³C-enriched DNA fractions of a previous DNA-stable isotope probing (DNA-SIP) experiment with soil from the same site [12]. Analogous to the single-substrate incubations, anoxic soil slurries were incubated for two months with low-amounts of sulfate and a ¹³C-labelled mixture of formate, acetate, propionate, and lactate. DNA was extracted, separated on eight replicated density gradients, and DNA from a total of 16 heavy fractions (density 1.715–1.726 g mL^−1^) was pooled for sequencing.

Additional DNA was obtained from soils that were sampled from different depths in the years 2004 and 2007 [13].

### Quantitative PCR and metagenome/-transcriptome sequencing

Abundances of *Acidobacteria* subdivision 1, 2, and 3 in soil samples from different years and depths were determined by newly-developed 16S rRNA gene-targeted real-time quantitative PCR (qPCR) assays (Supplementary Methods). Native soil DNA (two libraries), heavy ^13^C-enriched DNA (three libraries), and native soil RNA, and RNA samples from the microcosms were sequenced on an Illumina HiSeq 2000/2500 system (Supplementary Methods).

### Binning, phylogeny, and annotation of DsrAB-encoding genomes

The differential coverage binning approach by Albertsen et al. [16] was applied to extract MAGs of interest. The raw FASTQ paired-end reads were imported into the CLC Genomics Workbench 5.5.1 (CLC Bio) and trimmed using a minimum Phred quality score of 20 with no ambiguous nucleotides allowed. TruSeq adapters were removed and a minimum length filter of 50 nt was applied. This resulted in 214, 171, 233, 49, and 294 million reads after quality filtering and trimming for the two native soil and three SIP metagenomes, respectively (84–95% of the raw reads). All reads were co-assembled using CLCs de novo assembly algorithm (kmer size 63, bubble size 50, minimum scaffold size 1000 nt). The reads from all five metagenomes were independently mapped to the assembled scaffolds using CLCs map to reference function (minimum length 0.7, minimum similarity 0.95) to obtained the scaffold coverage. The SIP metagenomes were merged into one mapping. 137, 112, and 376 million reads could be mapped to the two native soil metagenomes and the SIP metagenome, respectively (64–66% of quality filtered reads). Gene prediction of the complete assembly was performed using prodigal [17]. In addition to the detection and taxonomic classification of 105 essential marker genes [16], *dsrA* and *dsrB* homologs were identified using TIGRFAM’s hidden Markov model (HMM) profiles TIGR02064 and TIGR02066, respectively, with HMMER 3.1 [18] and the provided trusted cut-offs. Additional *dsrAB*-containing scaffolds were identified by using tblastn with the published DsrAB database as a query against the assembly [3]. DsrAB sequences were classified by phylogenetic analysis (Supplementary Methods; [3]). Binning and decontamination was finalized utilizing the G+C content and tetramer frequencies of the scaffolds, as well as paired-end information, as described and recommended in Albertsen et al. [16]. Completeness, contamination, and strain heterogeneity was estimated using CheckM 1.0.6 [19] with lineage-specific marker sets selected at phylum rank (or class rank for *Proteobacteria*). MAGs were taxonomically classified by phylogenomic analysis of concatenated marker sequences and calculation of average nucleic and amino acid identities (ANI, AAI, Supplementary Methods). MAGs were annotated using the MicroScope annotation platform [20] and eggNOG [21]. Genes of interest (Supplementary Table S2) were manually curated using the full range of tools integrated in MicroScope annotation platform (Supplementary Methods).

### Genome-centric activity analysis: iRep and metatranscriptomics

The index of replication (iRep) was calculated for each MAG with the combined native soil metagenomes. Settings and thresholds were applied as recommended [22] using bowtie2 [23] and the iRep script with default settings. Quality-filtered metatranscriptome reads were mapped to all genomes using bowtie2 and counted with featureCounts [24]. To determine gene expression changes, we applied the DESeq2 pipeline with recommended settings [25] (Supplementary Methods).

### Data availability

Metagenomic and -transcriptomic data were deposited under the BioProject accession numbers PRJNA412436 and PRJNA412438, respectively, and can also be obtained via the JGI’s genome portal (JGI Proposal ID 605). MAGs are available at MicroScope (https://www.genoscope.cns.fr/agc/microscope/) and were deposited at the European Nucleotide Archive (PRJEB24926). DsrAB sequences were deposited at NCBI GenBank under the accession numbers MG182080–MG182141.

## Results

### Functional metagenomics: recovery of *dsrAB*-containing genomes from native soil and ¹³C-DNA fraction metagenomes

**Fig. 1:**
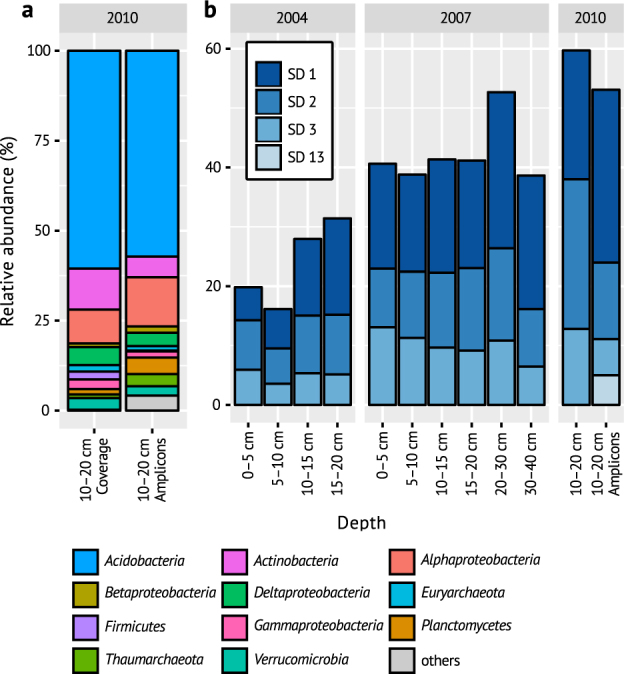
Microbial community composition in Schlöppnerbrunnen II peatland in samples from different years and soil depths. **a** Abundance of phyla and proteobacterial classes in the native soil (relative to all classified reads/amplicons). Taxa less abundant than 1% are grouped in grey. Coverage abundance is based on metagenomic reads mapped to classified scaffolds. Amplicon abundance is based on *rrn* operon-copy number-corrected abundance of 16S rRNA gene operational taxonomic units [9]. **b** Relative abundance of acidobacterial subdivisions (SD) in the native soil samples as determined by 16S rRNA gene qPCR assays. In addition, all subdivisions more abundant than 1% in a 16S rRNA gene amplicon dataset are shown [9]

This study was conducted with soil samples from the Schlöppnerbrunnen II peatland in Germany, which is a long-term study site with active sulfur cycling and harbors a large diversity of unknown microorganisms with divergent *dsrAB* genes [5, 13]. We initially generated co-assembled metagenomes from native peat soil DNA (53 Gb) and a pool of DNA extracts from the heavy fractions of a previous DNA-stable isotope probing (DNA-SIP) experiment with soil from the same peat (101 Gb). The heavy fractions, which were obtained from anoxic peat incubations with periodically supplemented sulfate and a mixture of ¹³C-labelled formate, acetate, propionate, and lactate at low concentrations, were enriched in DNA from *Desulfosporosinus* and also harbored DNA from yet unidentified *dsrAB*-containing microorganisms [12]. Based on the metagenome data, the native peat was dominated by *Acidobacteria* (61%), but also had *Actinobacteria*, *Alphaproteobacteria*, and *Deltaproteobacteria* as abundant (>5%) phyla/classes (Fig. 1). Dominance of *Acidobacteria*, *Alpha-* and *Deltaproteobacteria* is typical for peatlands [26]. Quantitative PCR confirmed that *Acidobacteria* subdivisions 1, 2, and 3 persistently dominated the Schlöppnerbrunnen II peat microbiota in oxic and anoxic soil layers (Supplementary Methods, Fig. 1), as observed in other peatlands [27–29].

We identified 36 complete or partial *dsrAB* genes on scaffolds of the co-assembled metagenome and subsequently recovered thirteen MAGs of DsrAB-encoding bacteria by differential coverage binning (Supplementary Table S1, [16]). Twenty-eight *dsrAB* sequences were part of the reductive bacterial-type DsrAB family branch and were closely related to previously recovered sequences from this and other wetlands (Supplementary Figure S1). These *dsrAB* sequences were affiliated to the known SRM genera *Desulfosporosinus* (*Firmicutes*, *n* = 1, one MAG) and *Syntrophobacter* (*Deltaproteobacteria*, *n* = 3, two MAGs), the *Desulfobacca acetoxidans* lineage (*n* = 1), and the uncultured DsrAB family-level lineages 8 (*n* = 19, seven MAGs) and 10 (*n* = 4). Six sequences grouped with the oxidative bacterial-type DsrAB family and were distantly affiliated with *Sideroxydans lithotrophicus* (*Betaproteobacteria*, *n* = 5, two MAGs) or *Rhodomicrobium vannielii* (*Alphaproteobacteria*, *n* = 1) (Supplementary Figure S2). Interestingly, two of our sequences (*n* = 2, one MAG) and a DsrAB sequence from the candidate phylum *Rokubacteria* [30] formed a completely novel basal lineage outside the four previously recognized DsrAB enzyme families (Supplementary Figure S2) [3]. The thirteen partial to near complete *dsrAB*-containing MAGs had moderate to no detectable contamination as assessed by CheckM and manual curation (Supplementary Table S1) [19] and derived from *Acidobacteria* subdivisions 1 and 3 (SbA1–7), *Desulfosporosinus* (SbF1), *Syntrophobacter* (SbD1, SbD2), *Betaproteobacteria* (SbB1, SbB2), and *Verrucomicrobia* (SbV1), as inferred by phylogenetic analysis of DsrAB sequences (Supplementary Figures S1 and S2) and concatenated sequences of single-copy, phylogenetic marker genes (Supplementary Figure S3). Only the *Desulfosporosinus* and *Syntrophobacter* MAGs contained rRNA gene sequences.

Phylogenomic analysis showed that *Acidobacteria* MAGs SbA1, SbA5, and SbA7 are affiliated with subdivision 1, while SbA3, SbA4, and SbA6 are affiliated with subdivision 3 (Supplementary Figure S3). The partial MAG SbA2 lacked the marker genes used for phylogenomic treeing, but was unambiguously assigned to *Acidobacteria* using an extended marker gene set [16] and DsrAB phylogeny. The two near complete (96%) MAGs SbA1 and SbA5 have a size of 5.4 and 5.3 Mb, respectively. The G+C content of all acidobacterial MAGs ranges from 58 to 63% (Supplementary Table S1). This in accordance with genome characteristics of acidobacterial isolates, which have genome sizes of 4.1–10.0 Mb and G+C contents of 57–62% [31, 32]. SbA1 and SbA7 form a monophyletic clade in the Acidobacteria subdivision 1 with an AAI [33] of 63% (Supplementary Figure S3) and DsrAB identity of 80% as was calculated with T-Coffee 11 [34] using the unfiltered reference alignment without the intergenic region [3]. They have 56% AAI to their closest relative, *Ca. Koribacter versatilis*, which is lower than AAIs among members of known acidobacterial genera (60–71%). The third MAG from subdivision 1, SbA5, is affiliated with *Terracidiphilus gabretensis* with an AAI of 61%. DsrAB identity of SbA5 to SbA1 and SbA7 is 79%. The three subdivision 3 MAGs form a monophyletic clade with *Ca. Solibacter usitatus* (Supplementary Figure S3). SbA3, SbA4, and SbA6 have AAIs of 59–73% amongst them and 61–62% to *Ca. S. usitatus*. DsrAB identity amongst the three MAGs is 80–94% and 74–79% to the subdivisions 1 MAGs.

The DsrAB sequences encoded on all seven MAGs belong to the uncultured DsrAB family-level lineage 8 (Supplementary Figure S1), which so far only consisted of environmental *dsrAB* sequences of unknown taxonomic identity [3]. Based on these MAGs and metatranscriptome analyses of anoxic peat soil microcosms, we describe the putative metabolic capabilities of these novel DsrAB-encoding *Acidobacteria*. Details on the other MAGs will be described elsewhere (Hausmann et al., unpublished; Anantharaman et al., unpublished). Functional interpretations of the recovered MAGs are made under the premise that the genomes are not closed, and thus it is unknown if genes are absent in these organisms or are missing due to incomplete sequencing, assembly, or binning.

### Dissimilatory sulfur metabolism

**Fig. 2:**
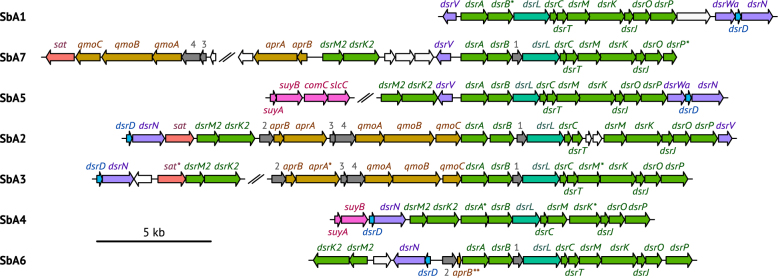
Organization of dissimilatory sulfur metabolism genes on acidobacterial MAGs SbA1–7. Red: *sat*; orange: *aprBA*, *qmoABC*; green: *dsrABCMKJOPM2K2*; blue: *dsrD*; turquoise: *dsrL*; violet: *dsrNVWa*; pink: *suyAB*, *comC*, *slcC*; 1–4 (grey): syntenic genes encoding for conserved proteins of unknown function; white: genes of unknown function or not involved in sulfur metabolism. In SbA2 all genes are on one scaffold (scaffold 0lkb). Gene fragments at contig borders are indicated by an asterisk. *aprB* in SbA6, indicated by two asterisks, is truncated, which indicates a pseudogene or is due to an assembly error. Scaffolds are separated by two slashes

Although *Acidobacteria* are abundant in diverse environments with active sulfur cycling [28, 29, 35, 36], this is the first discovery of members of this phylum with a putative dissimilatory sulfur metabolism. SbA2, SbA3, and SbA7 encode the complete canonical pathway for dissimilatory sulfate reduction, including homologs for sulfate transport (*sulP* and/or dass, not in SbA7) and activation (*sat*, ppa, *hppA*), adenosine 5′-phosphosulfate (APS) reduction (*aprBA*, *qmoABC*), and sulfite reduction (*dsrAB*, *dsrC*, *dsrMKJOP*) (Fig. 2, Supplementary Table S2a) [37]. In the AprBA tree, the acidobacterial sequences are part of a large cluster of yet uncultured organisms and *Deltaproteobacteria* and *Firmicutes* that respire sulfate, sulfite, or thiosulfate (Supplementary Figure S4) [4]. SbA1, SbA4, SbA5, and SBA6 have an incomplete sulfate reduction gene set but contain all *dsr* genes for sulfite reduction. Several other *dsr* genes were present on some of the MAGs. The *dsrD* and *dsrN* genes occurred in pairs. The acidobacterial DsrD sequences have the same conserved, hydrophobic residues as *Desulfovibrio vulgaris* DsrD (Supplementary Figure S5) [38]. Ubiquity of DsrD among SRM suggests an essential function in sulfate reduction, but the physiological role of this small protein is unresolved [39]. DsrN is a homolog of cobyrinate a,c-diamide synthase in cobalamin biosynthesis and may be involved in amidation of the siroheme prosthetic group of DsrAB [40]. DsrV, a homolog of precorrin-2 dehydrogenase, and DsrWa, a homolog of uroporphyrin-III C-methyltransferase, may also be involved in siroheme biosynthesis [41]. DsrT is required for sulfide oxidation in *Chlorobaculum tepidum*, but also found in SRM [41]. The presence of *dsrMK*-paralogs (*dsrM2*, *dsrK2*) upstream of *dsrAB* is not uncommon in SRM [42]. DsrMK are present in all *dsrAB*-containing microorganisms and are a transmembrane module involved in reduction of cytoplasmic DsrC-trisulfide in SRM, the final step in sulfate reduction [37]. DsrC encoded on the MAGs have the two essential cysteine residues at the C-terminal end for full functionality [43]. Interestingly, *dsrC* forms a gene duo with *dsrL* downstream of *dsrAB* in all seven MAGs. This is surprising, because *dsrL* is not found in SRM but in sulfur oxidizers. DsrL is highly expressed and essential for sulfur oxidation by the purple sulfur bacterium *Allochromatium vinosum* [40, 44]. DsrL is a cytoplasmic iron-sulfur flavoprotein with proposed NAD(P)H: acceptor oxidoreductase activity and was copurified with DsrAB from the soluble fraction of *A. vinosum* [45]. The acidobacterial DsrL sequences are shorter than their homolog in *A. vinosum* (Supplementary Table S2a), but have the same functional domains (Supplementary Figure S6). Given the possible role of DsrL in sulfur oxidation, we sought to detect additional genes indicative of oxidative sulfur metabolism in the acidobacterial MAGs. However, genes for Sox enzyme machinery (*soxABXYZ*), thiosulfate dehydrogenase (*tsdA*), sulfide:quinone reductase (*sqr*), adenylyl-sulfate reductase membrane anchor subunit (*aprM*), flavocytochrome c sulfide dehydrogenase (*fccAB*), sulfur reductase (*sreABC*), thiosulfate reductase (*phsABC*), polysulfide reductase (*psrABC*), membrane-bound sulfite oxidizing enzyme (*soeABC*), cytoplasmic sulfur trafficking enzymes (*tusA*, *dsrE2*, *dsrEFH*), or DsrQ/DsrU (unknown functions) were absent [1, 41, 46, 47]. SbA1, SbA3, SbA4, and SbA6 contain genes that have only low homology to *soxCD*/*sorAB*, periplasmic sulfite-oxidizing enzymes (Supplementary Results) and, thus, might have another function [48].

Despite ongoing sulfur cycling, concentrations of inorganic sulfur compounds such as sulfate are low (lower µM range) in the Schlöppnerbrunnen II peatland [49–51]. Enzymatic release of inorganic sulfur compounds from organic matter might thus represent a significant resource for sulfur-dissimilating microorganisms. Therefore, we specifically searched for genes coding for known organosulfur reactions that yield sulfite [1]. Genes for cysteate sulfo-lyase (*cuyA*), methanesulfonate monooxygenase (*msmABCD*), sulfoacetaldehyde acetyltransferase (xsc), and taurine dioxygenase (*tauD*) were absent. However, *suyAB*, coding for the (*R*)-sulfolactate sulfo-lyase complex that cleaves (*R*)-sulfolactate into pyruvate and sulfite [52], were present in SbA4 and SbA5 (Supplementary Table S2a). Intriguingly, SbA4 and SbA5 only have capability for sulfite reduction. SbA5 also encodes the racemase machinery for (*S*)-sulfolactate to (*R*)-sulfolactate, (*S*)-sulfolactate dehydrogenase (*slcC*) and (*R*)-sulfolactate dehydrogenase (*comC*); the regulator gene *suyR* or the putative importer SlcHFG were absent [52]. Pyruvate may be used as an energy and carbon source, while sulfite could be used as an electron acceptor for anaerobic respiration [53].

### Respiration

**Fig. 3:**
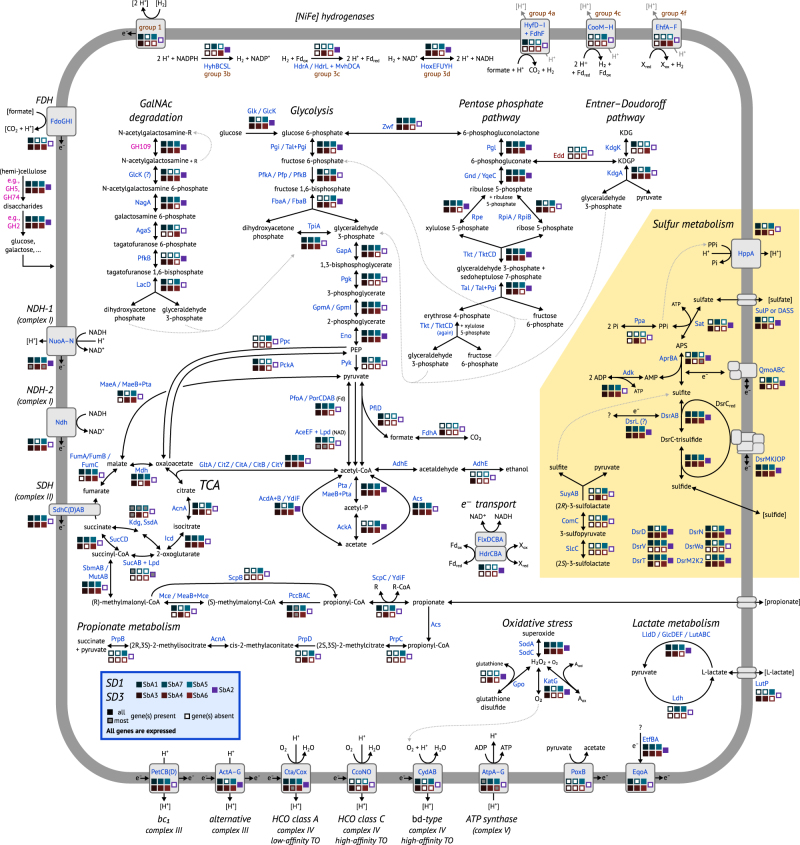
Metabolic model as inferred from analysis of acidobacterial MAGs SbA1–7. Sulfur metabolism is highlighted in yellow. Enzymes and transporters are shown in blue font. Glycoside hydrolases are shown in pink font (Supplementary Table S2). Extracellular compounds are in parentheses. A slash (/) indicates isozymes, i.e., enzymes that perform the same function, but are distinctly different or have more than one established name. AcdA+B, MaeB+Pta, MeaB+Mce, Tal+Pgi: bifunctional fusion genes/proteins. Otherwise the plus sign (+) indicates protein complexes. TCA: tricarboxylic acid cycle, FDH: formate dehydrogenase, Hase: hydrogenase, NDH: NADH dehydrogenase, HCO: haem-copper oxidase, TO: terminal oxidase, KDG: 2-dehydro-3-deoxy-D-gluconate, KDGP: 2-dehydro-3-deoxy-D-gluconate 6-phosphate. Expression of at least one copy of every enzyme and transporter was observed in the incubation samples

Cultivated *Acidobacteria* of subdivisions 1 and 3 are strict aerobes or facultative anaerobes (e.g., [54–59]). Accordingly, we found respiratory chains encoded in all acidobacterial MAGs (Fig. 3, Supplementary Results), with (near) complete operons for NADH dehydrogenase 1, succinate dehydrogenase (lacking in SbA2), one or both types of quinol—cytochrome-c reductase, low-affinity terminal oxidases, and ATP synthase (lacking in SbA2) (Supplementary Tables S2b–h). High-affinity terminal oxidases, putatively involved in detoxification of oxygen [60, 61], are limited to four MAGs (Supplementary Table S2g). Genes for dissimilatory nitrogen or iron metabolisms are absent, with the exception of a putative metal reductase in SbA2 of unclear physiological role (Supplementary Results).

### Hydrogen utilization and production

We identified [NiFe] hydrogenases of groups 1, 3, and 4 [62] in SbA1–7 (Supplementary Table S2j). Membrane-bound group 1 hydrogenases (SbA1, SbA3, SbA5) consume hydrogen from the periplasm as an electron donor to generate energy, possibly coupled to sulfate/sulfite reduction. In contrast to other *Acidobacteria*, no group 1h/5 hydrogenases, which are coupled to oxygen respiration, were identified [63]. Cytoplasmic group 3 hydrogenases (all MAGs) are bidirectional and proposed to be involved in energy-generating hydrogen oxidation and/or fermentative hydrogen production. Membrane-bound group 4 hydrogenases (SbA1, SbA5, SbA4, SbA6) produce H_2_ and are postulated to conserve energy by proton translocation by oxidizing substrates like formate (group 4a) or carbon monoxide (via ferredoxin, group 4c) (Fig. 3).

### A versatile heterotrophic physiology

**Fig. 4:**
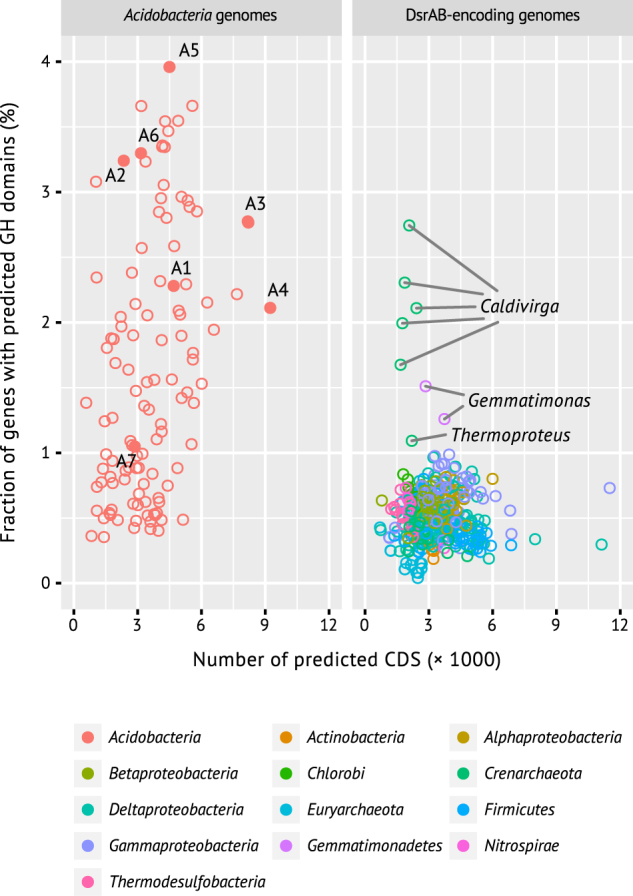
Glycoside hydrolase genes are enriched in acidobacterial genomes/MAGs compared to genomes from other taxa that encode DsrA/DsrB. DsrAB-containing MAGs SbA1–7 are shown as solid symbols and numbered accordingly. X-axis shows the total number of predicted CDS per genome/MAG

*Acidobacteria* are known for their capability to degrade simple and polymeric carbohydrates [31, 55–59, 64, 65], supported by many diverse carbohydrate-active enzymes encoded on their genomes [31, 32]. Accordingly, the MAGs recovered in our study also contain many genes encoding diverse carbohydrate-active enzymes (Supplementary Methods, Fig. 4). These include glycoside hydrolases (GH, 1.0–4.0% of all genes), polysaccharide lyases (0.07–0.3%), and carbohydrate esterases (0.7–1.4%) that are generally involved in degradation of complex sugars, but also glycosyltransferases (0.9–1.4%) for biosynthesis of carbohydrates. Functional GH families (assigned by EC number) putatively involved in cellulose and hemicellulose degradation were most prevalent (Supplementary Table S4). Specifically, the most often encountered EC numbers encompassed by the different GH families represented cellulose (EC 3.2.1.4, e.g., GH5, GH74), xyloglucan (EC 3.2.1.150, EC 3.2.1.151, e.g., GH5, GH74), or xylan (EC 3.2.1.8, EC 3.2.1.37, e.g., GH5) degradation, which is similar to the situation found in other members of *Acidobacteria* subdivision 1 and 3 [31, 32]. Further EC numbers that were often encountered in the various detected GH families were associated with oligosaccharide degradation (EC 3.2.1.21, e.g., GH2) or α-*N*-acetylgalactosaminidase activity (EC 3.2.1.49, e.g., GH109). Degradation of cellulose and hemicellulose yields glucose and all MAGs encode glycolysis and pentose phosphate pathways (Fig. 3, Supplementary Results). α-*N*-acetylgalactosaminidase releases *N*-acetylgalactosamine residues from glycoproteins that are commonly found in microbial cell walls and extracellular polysaccharides [66]. *N*-acetylgalactosamine can not be directly utilized via glycolysis, however the additionally required enzymes are present (Fig. 3; Supplementary Results). Under oxic conditions, organic carbon could be completely oxidized to CO_2_ via the citric acid cycle (Fig. 3). Alternatively, we also identified fermentative pathways. SbA3 encodes the bifunctional aldehyde-alcohol dehydrogenase AdhE that yields ethanol (Fig. 3). All MAGs encode additional aldehyde and alcohol dehydrogenases without clear substrate specificity that could also ferment acetyl-CoA to ethanol. SbA7 and SbA5 encode a L-lactate dehydrogenase (Ldh) yielding lactate from pyruvate, while six MAGs encode L-lactate dehydrogenases (LldD, GlcDEF, LutABC) that presumably perform the reverse reaction (Fig. 3). Similarly, we identified pathways for acetate and/or propionate production or utilization in all MAGs (Fig. 3; Supplementary Results). SbA1 and SbA3 potentially produce H_2_ via formate C-acetyltransferase PflD, which cleaves pyruvate into acetyl-CoA and formate. SbA1 encodes for the membrane-bound formate hydrogenlyase complex (*fdhF*, *hyf* operon) that produces H_2_ and might also translocate protons. SbA3 harbours an uncharacterized, cytoplasmic, monomeric FDH (*fdhA*) to transform formate to H_2_. SbA1, SbA3, SbA4, and SbA6 also encode membrane-bound, periplasmic FDH (*fdo* operon) that transfers electrons into the membrane quinol pool, as a non-fermentative alternative of formate oxidation (Fig. 3, Supplementary Table S2j).

### DsrAB-encoding *Acidobacteria* are metabolically active under anoxic conditions

We calculated the iRep based on the native peat soil metagenomes to assess whether DsrAB-encoding *Acidobacteria* were active in situ [22]. SbA1 and SbA5, which were sufficiently complete (≥75%) for a reliable estimate, had iRep values of 1.21 and 1.19, respectively. This shows that a fraction of each population was metabolically active, i.e., on average 21% of SbA1 and 19% of SbA5 cells were actively replicating at the time of sampling. Concordantly, SbA1–7 were also transcriptionally active in the same native soil samples. 35–46% of the SbA1–7 genes were expressed in at least one replicate. SbA1 and SbA5 contributed a considerable fraction (0.4% and 1.8%, respectively, Supplementary Table S1) of the total mRNA reads in the native soil metatranscriptome. These data likely underestimate the metabolic activity of SbA1–7 in situ because freshly sampled soil was stored at 4 °C for one week prior to nucleic acids extraction.

We further analyzed metatranscriptome data from a series of anoxic incubations of the peat soil with or without individual substrates (formate, acetate, propionate, lactate, or butyrate) and with or without supplemental sulfate [9]. While the incubations were not designed to specifically test for the MAG-inferred metabolic properties, they still allowed us to evaluate transcriptional response of the DsrAB-encoding *Acidobacteria* under various anoxic conditions (Supplementary Methods and Results). All treatments triggered shifts in genome-wide gene expression; more genes were significantly (*p* < 0.05) upregulated (73–933) than downregulated (14–81) as compared to the native soil. Upregulated genes included sulfur metabolism enzymes, high-affinity terminal oxidases, group 1 and 3 hydrogenases, aldehyde-alcohol dehydrogenase AdhE, glycoside hydrolases, and other carbon metabolism enzymes (Supplementary Table S3, Supplementary Figure S7). Significantly upregulated glycoside hydrolase genes belonged to GH family 2, 3, 5, 9, 10, 18, 20, 23, 26, 28, 29, 30, 33, 35, 36, 38, 43, 44, 50, 51, 55, 74, 76, 78, 79, 88, 95, 97, 105, 106, 109, and 129 in MAGs SbA1–6. None of the GH genes were significantly downregulated in the incubations. Noteworthy genes that were significantly downregulated were superoxide dismutases (*sodA*) in SbA2 and SbA4 (Supplementary Table S3a).

## Discussion

Diverse members of the phylum *Acidobacteria* are abundant in various ecosystems, particularly in soils and sediments with relative abundances typically ranging from 20–40% [67]. *Acidobacteria* are currently classified in 26 subdivisions based on their 16S rRNA phylogeny [68]. Given their phylogenetic breadth, comparably few isolates and genomes are available to explore their metabolic capabilities. Currently isolated species of subdivisions 1, 3, 4, and 6 are aerobic chemoorganotrophs that grow optimally at neutral or low pH [26, 64, 65, 69]. Furthermore, subdivision 4 contains an anoxygenic phototroph [70, 71], subdivisions 8 and 23 contain anaerobes [72–74], subdivisions 1, 3, and 23 fermenters [56, 59, 73, 75] and subdivision 4, 8, 10, and 23 thermophiles [71, 73, 76, 77].

*Acidobacteria* are known as dominant inhabitants of wetlands worldwide, in particular members of subdivision 1, 3, 4, and 8 [26]. Strains in the genera *Granulicella* [57], Telmatobacter [59], Bryocella [58] and *Bryobacter* [55] have been isolated from acidic wetlands and are presumably active in plant-derived polymer degradation (such as cellulose) [26, 78–80], and in nitrogen and iron cycling [50, 56].

Here, we provide metagenomic and metatranscriptomic evidence that the newly discovered species represent at least three novel genera in *Acidobacteria* subdivision 1 and 3 (Supplementary Figure S3) and possess a dissimilatory sulfur metabolism. The seven acidobacterial MAGs from the Schlöppnerbrunnen II peatland encode the complete canonical pathway for dissimilatory sulfite or sulfate reduction. The sulfate reduction pathway, however, could also operate in reverse as proposed for a sulfur-oxidizing deltaproteobacterium [81]. The phylogenetic separation into two subdivisions as based on the concatenated marker gene tree is also apparent in the DsrAB phylogeny (Supplementary Figure S1). The acidobacterial DsrAB sequences are distributed among two monophyletic clades within the uncultured family-level lineage 8, which is part of the reductive, bacterial-type DsrAB branch [3]. The phylogenetic breadth of the acidobacterial DsrAB sequences is representative for the intra-lineage sequence divergence within uncultured DsrAB lineage 8, which suggests that this entire lineage represents yet uncultivated bacteria of the phylum *Acidobacteria*. Members of this uncultured DsrAB lineage are widespread in freshwater wetlands (Supplementary Figure S1) [5]. In particular, they represented an abundant fraction of the DsrAB diversity and were permanent autochthonous inhabitants of oxic and anoxic soil layers in the analyzed Schlöppnerbrunnen II peatland [12, 13].

Presence of the complete gene set for canonical dissimilatory sulfate reduction suggests that the pathway is functional, as the genetic capability for sulfate reduction can be rapidly lost by adaptive evolution if unused [82]. Except for a truncated *aprB* on SbA6, we found no indications of pseudogenes, i.e., unexpected internal stop codons or reading frame shifts, for any of the sulfate/sulfite reduction genes on the acidobacterial MAGs [3]. In addition, sulfur metabolism genes of each MAG were expressed in the native soil and the anoxic microcosms (Supplementary Table S3a). Many sulfur metabolism genes were even significantly upregulated in the anoxic microcosms, with *dsrC* and *aprBA* among the top 10 most expressed genes in SbA7 (Supplementary Table S3a). These findings further support full functionality of the acidobacterial dissimilatory sulfur pathways under anoxic condition.

Known SRM typically couple sulfate respiration to oxidation of fermentation products such as volatile fatty acids, alcohols, or hydrogen [83]. While other microorganisms in the Schlöppnerbrunnen II soil, such as *Desulfosporosinus*, showed sulfate-specific and substrate-specific responses in our microcosms, hundreds of acidobacterial 16S rRNA phylotypes did not (with the exception of two) [9]. Gene expression patterns of DsrAB-encoding *Acidobacteria* in the individual anoxic microcosms as analyzed in the present study were ambiguous. Genes for putative oxidation of the supplemented substrates (formate, acetate, propionate, lactate, butyrate) were not specifically upregulated, neither without nor with supplemental sulfate. However, sulfur metabolism genes were upregulated in several incubations as compared to no-substrate-controls, suggesting indirect stimulation of a sulfur-based metabolism (Supplementary Results, Supplementary Table S3a). Indirect changes in microbial activity after the addition of fresh organic matter is often observed in soils (priming effects, [84]). One explanation for this priming effect is the co-metabolism theory stating that easily available substrates provide the energy for microorganisms to produce extracellular enzymes to make immobile carbon accessible, which is then also available to other microorganisms. The DsrAB-encoding *Acidobacteria* have a large genetic repertoire to utilize carbohydrates and monomeric sugars (Fig. 3). This is in accordance with the carbohydrate utilization potential previously described for subdivision 1 and 3 *Acidobacteria* [31, 32]. Yet utilization of monomeric sugars is a rare feature of known SRM [85, 86] and utilization of polysaccharides or oligosaccharides by sulfate-reducing bacteria was not yet reported. While the studied *Acidobacteria* expressed many of their glycoside hydrolase genes in our anoxic peat soil microcosms, further experiments are required to confirm if DsrAB-encoding *Acidobacteria* couple degradation of carbohydrate polymers or monomers to sulfate reduction.

It is intriguing to propose that MAGs SbA2, SbA3, and SbA7 derive from acidobacterial SRM as they lack known sulfur oxidation genes, except *dsrL* (Supplementary Figure S6), and express the complete dissimilatory sulfate reduction pathway (Supplementary Table S2a), including reductive, bacterial-type *dsrAB*, and *dsrD* that may be exclusive to SRM [39, 48, 87, 88]. However, the functions of DsrL and DsrD are yet unresolved, which prevents functional predictions based only on these genes. The proposal of an alternative hypothesis that these novel *Acidobacteria* reverse the sulfate reduction pathway for dissimilatory sulfur oxidation or sulfur disproportionation, bases on findings with the deltaproteobacterium *Desulfurivibrio alkaliphilus* [81]. *D. alkaliphilus* also lacks known sulfur oxidation genes (including *dsrL*), except for *sqr*, and is proposed to gain energy by coupling sulfide oxidation via a reversed sulfate reduction pathway (with a reductive-type DsrAB) to the dissimilatory reduction of nitrate/nitrite to ammonium. Sulfide oxidation in acidobacterial MAGs SbA2, SbA3, and SbA7 could proceed analogous to the pathway models proposed by Thorup et al. [81] and Christiane Dahl [89]. Briefly, hydrogen sulfide might react with DsrC either spontaneously [90] or via an unknown sulfur transfer mechanism to form persulfated DsrC. Persulfated DsrC is then oxidized by DsrMKJOP, thereby transferring electrons into the membrane quinone pool, and releasing a DsrC-trisulfide, which is the substrate for DsrAB [37, 89]. It was hypothesized that electrons released during DsrC-trisulfide oxidation to sulfite and DsrC are transferred to DsrL [89]. Further sulfite oxidation to sulfate would be catalyzed by AprBA-QmoABC and Sat.

The acidobacterial MAGs have the genomic potential to use oxygen as terminal electron acceptor and might thus couple sulfide oxidation to aerobic respiration. Alternative electron acceptors for biological sulfur oxidation in wetlands could include nitrate/nitrite and metals such as Fe(III) [50]. However, known genes for dissimilatory nitrate reduction and metal reduction [91] were absent from these acidobacterial MAGs. Only SbA2 encodes a putative metal reduction complex that was recently characterized in *Desulfotomaculum reducens* [92]. At this time, it is unclear whether DsrAB-encoding *Acidobacteria* are capable of Fe(III) respiration, as seen in *Geothrix fermentans* [74] and certain isolates in subdivision 1 [56, 93].

### Proposal of the acidobacterial *Candidatus* genera Sulfotelmatobacter, Sulfotelmatomonas, and Sulfopaludibacter

Based on combined interpretation of phylogeny (concatenated phylogenetic marker genes, DsrAB), genomic (ANI, AAI) and genetic (DsrAB) distances, and characteristic genomic features of dissimilatory sulfur metabolism (Fig. 3), in accordance with Konstantinidis et al. [94], we classify MAGs SbA1, SbA7, SbA5, SbA3, SbA4, and SbA6 into three new acidobacterial *Candidatus* genera, including *Candidatus* species names for the >95% complete MAGs SbA1 and SbA5. In-depth phylogenomic analysis of SbA2 was not possible and therefore it is tentatively assigned to *Acidobacteria* subdivision 3.

*Acidobacteria* subdivision 1

- *Ca*. genus Sulfotelmatobacter (Sul.fo.tel.ma.to.bac’ter. L. n. *sulfur*, sulfur; Gr. n. *telma*, *-tos*, swamp, wetland; N.L. masc. n. *bacter*, bacterium; N.L. masc. n. *Sulfotelmatobacter*, a bacterium from a swamp metabolizing sulfur) with *Ca*. Sulfotelmatobacter kueseliae MAG SbA1 (kue.se’li.ae. N.L. gen. n. *kueseliae*, of Kuesel, honouring Kirsten Küsel, for her work on the geomicrobiology of wetlands) and *Ca*. Sulfotelmatobacter sp. MAG SbA7.
- *Ca*. Sulfotelmatomonas gaucii MAG SbA5 (Sul.fo.tel.ma.to.mo.nas. L. n. *sulfur*, sulfur; Gr. n. *telma*, *-tos*, swamp, wetland; N.L. fem. n. *monas*, a unicellular organism; N.L. fem. n. *Sulfotelmatomonas*, a bacterium from a swamp metabolizing sulfur; gau’.ci.i. N.L. gen. n. *gaucii*, of Gauci, in honour of Vincent Gauci, for his pioneering work on the interplay of wetland sulfate reduction and global methane emission).

*Acidobacteria* subdivision 3

- *Ca*. genus Sulfopaludibacter (Sul.fo.pa.lu.di.bac’ter. L. n. *sulfur*, sulfur; L. n. *palus*, *-udis*, L. swamp; N.L. masc. n. *bacter*, bacterium; N.L. masc. n. *Sulfopaludibacter*, a bacterium from a swamp metabolizing sulfur) with *Ca*. Sulfopaludibacter sp. MAG SbA3, *Ca*. Sulfopaludibacter sp. MAG SbA4, and *Ca*. Sulfopaludibacter sp. MAG SbA6.
- *Acidobacteria* bacterium MAG SbA2.

## Conclusion

**Fig. 5:**
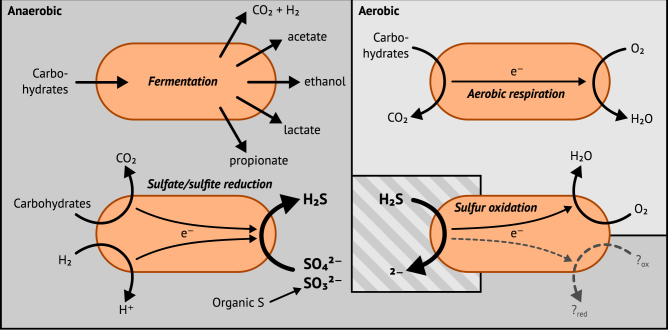
Putative lifestyles of DsrAB-encoding *Acidobacteria*

Sulfur cycling exerts important control on organic carbon degradation and greenhouse gas production in wetlands, but knowledge about sulfur microorganisms in these globally important ecosystems is scarce [5]. Here, we show by genome-centric metagenomics and metatranscriptomics that members of the phylum *Acidobacteria* have a putative role in peatland sulfur cycling. The genomic repertoire of these novel *Acidobacteria* species encompassed recognized acidobacterial physiologies, such as a facultative anaerobic metabolism, oxygen respiration, fermentation, carbohydrate degradation, and hydrogen metabolism, but was additionally augmented with a DsrAB-based dissimilatory sulfur metabolism (Fig. 5). Based on their genetic repertoire and previous findings on reversibility of the dissimilatory sulfate reduction pathway [81, 95–97], it is intriguing to speculate that the described peatland *Acidobacteria* could use the same pathway for both sulfate reduction and sulfide oxidation. The described DsrAB-carrying *Acidobacteria* that only encoded the pathway for dissimilatory sulfite reduction had additional genes for sulfite-producing enzymes, which suggests that organosulfonates might be their primary substrate for sulfur respiration. Our results not only extend the current understanding of the genetic versatility and distribution of dissimilatory sulfur metabolism among recognized microbial phyla, but also underpin the challenge to unambiguously differentiate between reductive or oxidative sulfur metabolism solely based on (meta-)genome/transcriptome data [81].

## Electronic supplementary material

Supplementary Methods, Results, Discussion, and Figures

Supplementary Tables
