# Supplementary material for: Peatland Acidobacteria with a dissimilatory sulfur metabolism
Source: ISME J. 2018 Feb 23;12(7):1729–42. doi: 10.1038/s41396-018-0077-1 (PMC6018796; doi:10.1038/s41396-018-0077-1)
Supplement: Supplementary file 1 — Supplementary Methods, Results, Discussion, and Figures [file 41396_2018_77_MOESM1_ESM.pdf]

Peatland *Acidobacteria* with a dissimilatory sulfur metabolism

Supplementary Information

Bela Hausmann, Claus Pelikan, Craig W. Herbold, Stephan Köstlbacher, Mads Albertsen, Stephanie A. Eichorst, Tijana Glavina del Rio, Martin Huemer, Per H. Nielsen, Thomas Rattei, Ulrich Stingl, Susannah G. Tringe, Daniela Trojan, Cecilia Wentrup, Dagmar Woebken, Michael Pester, Alexander Loy

Table of Contents

Supplementary Methods 1
Quantitative PCR analysis of acidobacterial subdivisions 1
Metagenomic and metatranscriptomic sequencing 1
Genome-based taxonomic classification and phylogeny 1
Manually curated annotation of of DsrAB-encoding genomes 2
Carbohydrate-active enzymes 2
Expression analysis 3
Supplementary Results and Discussion 3
Sulfite dehydrogenase homologs 3
Respiration and oxidative stress 3
Dissimilatory nitrogen metabolism and nitrogen fixation 5
Dissimilatory metal reduction 6
Import and phosphorylation of glucose 6
N-acetylgalactosamine degradation 6
Lactate, propionate, and butyrate metabolism 6
Differential gene expression between anoxic microcosm incubations 7
Supplementary References 8
Supplementary Tables 14
Supplementary Table S1 14
Supplementary Table S2 14
Supplementary Table S3 15
Supplementary Table S4 15
Supplementary Figures 16
Supplementary Figure S1 16
Supplementary Figure S2 18
Supplementary Figure S3 19
Supplementary Figure S4 20
Supplementary Figure S5 21
Supplementary Figure S6 22
Supplementary Figure S7 24

Supplementary Methods

Quantitative PCR analysis of acidobacterial subdivisions

Acidobacteria subdivisions 1, 2, and 3 were separately quantified using 16S rRNA gene-targeted real-time quantitative PCR (qPCR) assays. Coverage of the subdivisions was estimated using the RDP ProbeMatch online tool with RDP Release 11, Update 5, good quality filter applied, requiring a full match to the probe sequence (Cole *et al.*, 2014). The following parameters ensure optimized efficiency and sensitivity of each qPCR assay. Subdivision 1: Acid303Fa/Acid303Fb (5′-GCG CAC GGM CAC ACT GGA-3′/5′-GCG CGC GGC CAC ACT GGA-3′) and Acid657R (5′-ATT CCA CKC ACC TCT CCC AY-3′), 76%/0.1% coverage by primers pairs, primer concentration: 1000 nM, annealing temperature: 68.5 °C; Subdivision 2: Acid702Fa/Acid702Fb (5′-AGA TAT CTG CAG GAA CAY CC-3′/5′-AGA TAT CCG CAG GAA CAT CC-3′) and Acid805R (5′-CTG ATS GTT TAG GGC TAG-3′), 64%/7% coverage, primer concentration: 1000 nM, annealing temperature: 62.5 °C; Subdivision 3: Acid306F (5′-CAC GGC CAC ACT GGC AC-3′) and Acid493R (5′-AGT TAG CCG CAG CTK CTT CT-3′), 77% coverage, primer concentration: 500 nM, annealing temperature: 69 °C. Thermal cycling was carried out with an initial denaturation (94 °C) followed by 40–45 cycles of denaturation (94 °C, 40 s), annealing (68.5 °C, 62.5 °C, or 69 °C, 40 s), and elongation (72 °C, 40–45 s). PCR efficiency with perfectly-matched reference targets was between 82–86% with an R^2 of 0.99 and a limit of detection at 100 target genes per reaction. For calculation of relative abundances, total bacterial and archeal 16S rRNA genes were quantified using a previously published qPCR assay (Pester *et al.*, 2010; Hausmann *et al.*, 2016).

Metagenomic and metatranscriptomic sequencing

DNA was sent to the JGI, where it was fragmented to a target length of 270 nt. Libraries were generated with the KAPA-Illumina library creation kit (KAPA biosystems) and sequenced on an Illumina HiSeq2000 sequencer. The native soil yielded 232 million 150 nt paired-end reads. Three sequencing libraries of the pooled DNA-SIP sample yielded 273, 52, and 350 million 150 nt paired-end reads each. DNA from the native soil was also sent to the King Abdullah University of Science and Technology (Thuwal, Saudi Arabia), libraries prepared with the Nextera DNA Library Prep kit (Illumina), and sequenced on an Illumina HiSeq2000 sequencer (179 million 101 nt paired-end reads).

Triplicate RNA samples from the native soil and from each incubation treatment and time point were sent to the JGI. When possible, rRNA was depleted using Ribo-Zero rRNA removal kit (Epicentre). cDNA libraries were generated with the Truseq Stranded RNA LT kit (Illumina) and sequencing was performed on an Illumina HiSeq2000 sequencer. One propionate- and sulfate-stimulated replicate microcosm was excluded because of inconsistent response in sulfate turnover as compared to the other two replicates (Hausmann *et al.*, 2016), resulting in a total of 73 samples with 27–188 million 150 nt paired-end reads.

Genome-based taxonomic classification and phylogeny

Representative genome assemblies from the phylum *Acidobacteria* and outgroups from the *Proteobacteria*, *Firmicutes*, and *Verrucomicrobia* were obtained from NCBI for phylogenomic analysis. A filtered and concatenated amino acid alignment of 34 phylogenetically informative marker genes was created using CheckM (Parks *et al.*, 2015). Phylobayes was used to calculate the tree with a CAT-GTR model (Lartillot *et al.*, 2009). Phylobayes was run in five independent chains for 11000 cycles each (corresponding to approx. 6.8×10^6 tree generations per chain). The first 6000 cycles in each chain were discarded as burn in (corresponding to approx. 3.7×10^6 tree generations per chain).

Pairwise average nucleic and amino acid identities (ANI, AAI) between all protein-coding genes of each MAG and published reference genomes were calculated to estimate novelty (adapted from Varghese *et al.*, 2015). Two-way ANI and AAI were calculated based on reciprocal best blast hits filtered for sequence identity (≥70% and ≥30% for ANI and AAI, respectively) and alignment length (≥70% of the shorter sequence). Average identities and alignment fractions (AF) for each comparisons were calculated as outlined previously (Varghese *et al.*, 2015). None of the comparisons reached an ANI above the intra-species threshold of 96.5% (Varghese *et al.*, 2015). Due to lack of a generic intra-genus AAI threshold, we used the existing acidobacterial taxonomy as a reference. Intra-genus AAI variability of published acidobacterial genera with more than one species (*Acidobacterium*, *Granulicella*, *Terriglobus*) ranged from 60–71% (alignment fraction 52–66%).

Manually curated annotation of of DsrAB-encoding genomes

All genes of interest were manually curated using the MicroScope annotation platform (Vallenet *et al.*, 2017). This included assessment of best-BLAST-hits to reference genomes and UniProt entries (The UniProt Consortium, 2015), presence of the required functional domains (InterPro/InterProScan; Mitchell *et al.*, 2015; Jones *et al.*, 2014) and, if appropriate, transmembrane helices (TMhmm; Krogh *et al.*, 2001), membership in the correct COGs, and membership of syntenic regions (operons). COGs in the MicroScope annotation platform are assigned using COGnitor (www.ncbi.nlm.nih.gov/COG/) which are often too broad to be of use, therefore we additionally classified all coding DNA sequences (CDS) using the bactNOG database (eggNOG, Huerta-Cepas *et al.*, 2016). All possible HMM profiles were matched against every gene (E-value threshold 1) and only the best hit was extracted. This non-stringent E-value threshold allowed very small genes and fragments to be classified as well.

Carbohydrate-active enzymes

Carbohydrate-active enzymes (Lombard *et al.*, 2014, www.cazy.org) in the MAGs were identified and classified with dbCAN 4.0 (Yin *et al.*, 2012). For comparison, genomes belonging to the *Acidobacteria* and to genera with DsrAB-encoding members were downloaded from NCBI (June 2017) and analyzed with dbCAN as well. Genera with DsrAB-encoding members were identified based on literature research and UniProt InterPro/TIGRFAM searches for DsrA (IPR011806/TIGR02064) and DsrB (IPR011808/TIGR02066). For comparability and consistency, *de novo* ORF predictions were performed with prodigal (Hyatt *et al.*, 2010) for all genomes and MAGs. Presence of DsrA and/or DsrB was again verified with the TIGRFAM models and HMMER. dbCAN's HMM profiles were identified with HMMER and parsed with the provided dbCAN script and R (R Core Team, 2017). Analysed acidobacterial genomes belonged to the genera *Acidobacterium*, *Bryobacter*, *Chloracidobacterium*, *Edaphobacter*, *Geothrix*, *Granulicella*, *Holophaga*, *Koribacter*, *Luteitalea*, *Pyrinomonas*, *Silvibacterium*, *Solibacter*, *Terracidiphilus*, *Terriglobus*, and *Thermoanaerobaculum*. 82 additional acidobacterial genomes without genus classification were also analysed. DsrA/DsrB-encoding genomes derived from the genera *Acetonema*, *Achromatium*, *Acidiferrobacter*, *Alkalilimnicola*, *Allochromatium*, *Ammonifex*, *Anaeromyxobacter*, *Archaeoglobus*, *Azospirillum*, *Bilophila*, *Caldimicrobium*, *Caldivirga*, *Carboxydothermus*, *Chlorobaculum*, Chlorobium, *Curvibacter*, *Desulfacinum*, *Desulfamplus*, *Desulfarculus*, *Desulfatibacillum*, *Desulfatiglans*, *Desulfatirhabdium*, *Desulfatitalea*, *Desulfitibacter*, *Desulfitobacterium*, *Desulfobacca*, *Desulfobacter*, *Desulfobacterium*, *Desulfobacula*, *Desulfobulbus*, *Desulfocapsa*, *Desulfocarbo*, *Desulfococcus*, *Desulfocurvus*, *Desulfofervidus*, *Desulfofustis*, *Desulfohalobium*, *Desulfoluna*, *Desulfomicrobium*, *Desulfomonile*, *Desulfonatronospira*, *Desulfonatronovibrio*, *Desulfonatronum*, *Desulfonauticus*, *Desulfonispora*, *Desulfopertinax*, *Desulfopila*, *Desulfoplanes*, *Desulforegula*, *Desulforhopalus*, *Desulforudis*, *Desulfosarcina*, *Desulfospira*, *Desulfosporosinus*, *Desulfotalea*, *Desulfothermus*, *Desulfotignum*, *Desulfotomaculum*, *Desulfovermiculus*, *Desulfovibrio*, *Desulfovirgula*, *Desulfurella*, *Desulfurispora*, *Desulfurivibrio*, *Dethiosulfatarculus*, *Dissulfuribacter*, *Ferriphaselus*, *Gallionella*, *Gemmatimonas*, *Gordonibacter*, *Gracilibacter*,

Halodesulfovibrio, *Halorhodospira*, *Lamprocystis*, *Lautropia*, *Magnetococcus*, *Magnetomorum*, *Magnetoovum*, *Magnetospira*, *Magnetospirillum*, *Magnetovibrio*, *Marichromatium*, *Moorella*, *Pelodictyon*, *Phaeospirillum*, *Prosthecochloris*, *Pyrobaculum*, *Rhodomicrobium*, *Rubrivivax*, *Ruegeria*, *Ruthia*, *Sedimenticola*, *Sideroxydans*, *Sulfuricella*, *Sulfuritalea*, *Syntrophobacter*, *Syntrophomonas*, *Syntrophus*, *Thermanaeromonas*, *Thermocladium*, *Thermodesulfatator*, *Thermodesulfobacterium*, *Thermodesulfobium*, *Thermodesulforhabdus*, *Thermodesulfovibrio*, *Thermoproteus*, *Thermosinus*, *Thermosulfurimonas*, *Thioalkalivibrio*, *Thiobacillus*, *Thiocapsa*, *Thiocystis*, *Thiodiazotropha*, *Thioflavicoccus*, *Thioflexothrix*, *Thioglobus*, *Thiohalocapsa*, *Thiohalomonas*, *Thiolapillus*, *Thiomargarita*, *Thioploca*, *Thiorhodococcus*, *Thiorhodovibrio*, *Thiosymbion*, *Thiothrix*, and *Vulcanisaeta*.

Expression analysis

Metatranscriptomic reads were quality filtered at the JGI using their analysis pipeline. In short, the raw reads were quality-trimmed to Q10, adapter-trimmed using bbduk (minimum allowed length 50 nt), followed by removal of PhiX control sequences, artefacts, human sequences, and reads containing N bases with bbduk/bbmap (BBTools, http://jgi.doe.gov/data-and-tools/bbtools/). rRNA reads were removed using the SILVA database (Quast *et al.*, 2013) and bbmap. This resulted in 73 samples with 22–161 million high quality non-rRNA reads with a median length of 150 nt. Those were then mapped to the combined metagenomic assembly using bowtie2 with the default scoring function (Langmead and Salzberg, 2012). Fragments per CDS were then independently counted using featureCounts 1.5.0 (Liao *et al.*, 2014). Differential expression analysis of SbA1–7 was performed using R (R Core Team, 2017) and the DESeq2 package (Love *et al.*, 2014).

Supplementary Results and Discussion

Sulfite dehydrogenase homologs

We identified several genes encoding putative sulfite dehydrogenases of the COG2041 family: (A) SbA3 and SbA4 encode orthologs to *Cupriavidus necator* (Ralstonia eutropha) N-1 *sox*C (CNE_1c35220), SBA3_1920025 and SBA4_3730002, respectively (~50% sequence identity). Directly downstream are genes homologous to the N-terminal region of *C. necator soxD* (CNE_1c35210), SBA3_1920026 and SBA4_3730003, respectively (~40% sequence identity at <50% overlap). *sox*ABXYZ, present in *C. necator*, are not found on any acidobacterial MAG. However, *C. necator* N-1 can not oxidize thiosulfate (Dahl and Friedrich, 2008). (B) *Ca. Solibacter usitatus* encodes a *sor*AB-like gene pair (Acid_7248–7249), which we also found in SbA1 (SBA1_530003–4), SbA3 (SBA3_50016–17), SbA4 (SBA4_5130014–13 and fragmented SBA4_7680001–2), and SbA6 (SBA6_110054–55). The *sor*AB-like genes from the MAGs are <40% identical to *Starkeya novella* SorAB (Snov_3268–3269). SorA transfers electrons from oxidizing sulfite to the membrane-bound cytochrome c SorB subunit. SorB is then oxidized by a terminal oxidase. SorAB in *S. novella* is potentially involved in aerobic respiration or in sulfite detoxification (Simon and Kroneck, 2013). (C) YedYZ-like proteins are present in SbA1 (SBA1_880028–29), SbA4 (SBA4_240001–2, fragmented), and SbA5 (SBA5_120015–16), which are related to the sulfide oxidase family and are part of COG2041. Their function is unknown (Dahl and Friedrich, 2008) and they are found in several other acidobacterial genomes and *E. coli*. (D) Additional, completely uncharacterized members of COG2041 are present in SbA3, SbA4, and SbA5.

Respiration and oxidative stress

Respiration with oxygen as terminal electron acceptor requires a membrane electron transport chain involving up to four complexes, i.e., the NADH dehydrogenase (NDH, respiratory complex I), the

succinate dehydrogenase (SDH, respiratory complex II), the quinol–cytochrome-c reductase (cytochrome *bc*$_1$ complex or alternative complex III, respiratory complex III), and the terminal oxidase (cytochrome-c oxidase or cytochrome *bd*-type oxidase, respiratory complex IV). Complexes I (NDH-1 only), III, and IV (except *bd*-type) translocate protons through the cell membrane building up proton motive force. The ATP synthase uses the proton motive force to generate ATP and is called respiratory complex V. SbA5 and SbA7 encode every gene for complexes I–V, while SbA1, SbA3, SbA4, and SbA6 encode only partial operons for some complexes. SbA2, the most incomplete MAG, is lacking all genes of complex II and V (Figure 3).

Oxidation of organic matter generates reducing equivalents, e.g., in glycolysis one NADH is formed per one glucose. NADH is oxidized to NAD^+/H^+ by the NDH, which in turn transfers electron to the membrane quinone pool. Two types of NDH are characterized in *E. coli*. NDH-1, consisting of a large complex encoded by the *nuo* operon, translocates protons, while NDH-2, encoded by a single gene (*ndh*), does not. We identified both NDH-1 and NDH-2 in the MAGs, with the latter (partially) missing in SbA2, SbA4, and SbA7. All MAGs harbour one or more (partially fragmented) *nuo*ACDHJKLMN operons. One operon in each MAG also includes *nuoEFG* (except in SbA3). NuoEFG forms the catalytic NADH dehydrogenase module of complex I, while NuoBCDHIN and NuoKLM form the hydrogenase and transporter modules, respectively. NuoA and NuoJ are not part of the modules and likely involved in assembly of the complex (Friedrich *et al.*, 2016). SbA6 is missing *nuoI*, likely because of MAG incompleteness (Supplementary Table S2b).

SDH is encoded by the *sdh* operon. It consists of a cytoplasmic-facing catalytic subunit (SdhAB) and a transmembrane cytochrome b_{556} or b_{558} subunit (SdhCD in *E. coli* or larger SdhC in B. subtilis), which together transfer the electrons from oxidation of succinate to fumarate into the membrane quinone pool. It is the only complex of the respiratory chain not involved in proton translocation. Both complex I and II are found in many anaerobically respiring microorganisms, including SRM (e.g., Pereira *et al.*, 2011; Klenk *et al.*, 1997; Rabus *et al.*, 2004; Strittmatter *et al.*, 2009; Plugge *et al.*, 2012; Visser *et al.*, 2013; Kuever *et al.*, 2014; Mardanov *et al.*, 2016). We identified SDH in all MAGs but SbA2, arranged like the B. subtilis-type operon (*sdh*CAB) (Supplementary Table S2c). SbA1 harbours a second operon (*sdh*ACDB) that could also be a fumarate reductase (*frd*ACDB). Fumarate reductase performs the reverse reaction of SDH as part of the reductive citric acid cycle, but both enzymes were shown to catalyze both reaction in *E. coli* (Guest, 1981; Maklashina *et al.*, 1998). No other MAG harboured candidates for fumarate reductase.

Complex III, the quinol–cytochrome-c reductase transfers electron from the membrane quinone pool to cytochrome c. Cytochrome c is then oxidized and O_2 is reduced to H_2O by a cytochrome-c terminal oxidase. Alternately, a quinol terminal oxidase can directly utilize electrons from the membrane quinone pool to reduce O_2. Two isofunctional complexes of quinol–cytochrome-c reductases are known. The two component cytochrome *bc*$_1$ complex is encoded by the *pet* operon and present in all MAGs but SbA2 (Supplementary Table S2d). Alternative complex III (ACIII) (Refojo *et al.*, 2012) with seven subunits is encoded by the *act* operon and present in all MAGs but SbA7 (Supplementary Table S2e). Some of the terminal oxidase genes are found immediately down- or upstream of quinol–cytochrome-c reductase operons (Supplementary Tables S2e–g), as is observed in other *Acidobacteria* (e.g., *Ca. K. versatilis*, *Ca. S. usitatus*, *Chloracidobacterium thermophilum*; Garcia Costas *et al.*, 2012). In between alternative complex III and the terminal oxidase genes, we always find the *sco* gene coding for a chaperone of the SCO1/SenC family, a putative assembly factor for respiratory complexes (Buggy and Bauer, 1995). We identified both families of terminal oxidases, i.e., haem-copper oxidases (HCO) of classes A and C, which are cytochrome-c oxidases, and cytochrome *bd*-type oxidases, which are quinol oxidases. HCO class A are classified as low-affinity terminal oxidases (LATO) in contrast to HCO class C and *bd*-type oxidases that are high-affinity terminal oxidases (HATO) (Morris and Schmidt, 2013). Three distinct operon structures for HCO family A were observed in SbA1–7 and also other *Acidobacteria*, e.g. *Ca. K. versatilis* and *Ca. S. usitatus*: (1) downstream of ACIII

and *sco*, (2) upstream of *petBC* (not found in *Ca.* K. *versatilis*), both named *ctaCDEF*, and (3) without quinol–cytochrome-c reductase genes found up- or downstream but with a subunit III consisting of two genes (*coxOP*) (Supplementary Table S2f). The subunit II of all three types have the Cu_A copper center motifs (IPR001505), which is found in cytochrome-c oxidases but not in quinol oxidases (Pereira *et al.*, 2001). The essential subunits are I and II (Pereira *et al.*, 2001) and are present in all MAGs but SbA4. High-affinity terminal oxidases of HCO class C or *bd*-type oxidases are present in SbA1, SbA5, SbA3, and SbA6 (Supplementary Table S2g). Both types are encoded by two subunits on the MAGs. Secondary genes, as found in other organisms, e.g., *ccoQP* (Bühler *et al.*, 2010) or *cydS*/*cydX* (Cook and Poole, 2016), are missing.

An ATP synthase of the F_oF_1-ATPase type is present in all MAGs but SbA2 (Supplementary Table S2h). Its genes are consistently split into two operons, *atpZIBE* and *atpF′FHAGDC*. The former is missing in SbA6, while the later is fragmented in SbA1 with *atpG* missing completely. *atpF* and *atpF′* are paralogs of the subunit B. In cyanobacteria, a homodimer of subunit B is replaced by a heterodimer of subunit B and B′ (Dunn *et al.*, 2001). However, *atpF′F* is found in non-photosynthetic *Acidobacteria*, e.g., *Ca.* K. *versatilis* and *Ca.* S. *usitatus*, and also in the photoheterotroph *Chloracidobacterium thermophilum*.

A second function is attributed to terminal oxidases in some organisms, i.e., defence against oxidative stress, especially to *bd*-type oxidases (Giuffrè *et al.*, 2014). The strictly anaerobic SRM *Desulfovibrio vulgaris* encodes two terminal oxidases, one cytochrome-c oxidase (HCO class A, DVU_1815–1812) and one *bd*-type oxidase (DVU_3271–3270). It was demonstrated that both are involved in the detoxification of oxygen (Ramel *et al.*, 2013). Terminal oxidases are also needed to remove oxygen produced by superoxide detoxification. Superoxide dismutase converts superoxide to oxygen and hydrogen peroxide. Hydrogen peroxide is then removed by catalases, peroxidases, or glutathione peroxidases (Figure 3). Manganese-dependent superoxide dismutase (*sodA*) is found in all MAGs and Cu-Zn-dependent superoxide dismutase (*sodC*) in SbA2 and SbA7. SbA1, SbA2, SbA3, and SbA5 encode for bifunctional haem-dependent catalase-peroxidases (*katG*), but none of the seven MAGs for mono-functional, haem-dependent catalases (*katE*/*katA*) or manganese-dependent catalases (*katN*). SbA2 and SbA4 encode for glutathione peroxidases (Supplementary Table S2i).

Dissimilatory nitrogen metabolism and nitrogen fixation

Although nitrate availability is limited in wetlands (Pester *et al.*, 2012a), we investigated the possibility for nitrate respiration in the MAGs. SRM contribute to nitrogen cycling, as some can respire nitrate/nitrite as an alternative electron acceptor to sulfate or fix atmospheric nitrogen using nitrogenase (Rabus *et al.*, 2013; Marietou, 2016). Oxidation of sulfur compounds coupled to nitrate or nitrite reduction is common among sulfide-/thiosulfate-oxidizing microorganisms (Ghosh and Dam, 2009), but was also observed in organisms encoding reductive-type DsrAB genes. *Desulfovibrio desulfuricans*, *Desulfobulbus propionicus*, and *Desulfurivibrio alkaliphilus* were shown to oxidize sulfide with nitrate/nitrite as the electron acceptor (Dannenberg *et al.*, 1992; Thorup *et al.*, 2017). It is also proposed that the sulfide-oxidizing cable bacteria (*Desulfobulbaceae*) can use nitrate/nitrate as an alternative to oxygen (Marzocchi *et al.*, 2014).

Only few *Acidobacteria* were shown to perform nitrate reduction or encode the required marker genes (e.g., Ward *et al.*, 2009; Männistö *et al.*, 2012). SbA1–7 lack *narGHI*, *napAB*, *nrfA*, *nirK*, *nirS*, *norBC*, and *nosZ* (Kraft *et al.*, 2011) and thus the genomic potential for dissimilatory nitrate or nitrite reduction. Only SbA5 harbours two gene copies of the nitric oxide reductase NorZ (also known as qNOR), an enzyme that is likely not involved in denitrification but used for nitric oxide detoxification (Kraft *et al.*, 2011). The MAGs also lacked key genes of aerobic nitrogen metabolisms i.e., *amoCAB* (ammonia oxidation), *nxrAB* (nitrite oxidation), and *nifH* (nitrogen fixation) (Pester *et al.*, 2012b, 2014; Gaby and Buckley, 2014; Daims *et al.*, 2016).

Dissimilatory metal reduction

The genes required for dissimilatory metal reduction (*mtr/omc* operon) as described for *Shewanella* and *Geobacter* (Shi *et al.*, 2006; Weber *et al.*, 2006; Coursolle and Gralnick, 2010) are absent in all MAGs. Direct interspecies electron transfer (DIET) is an important but understudied process in wetlands (Holmes *et al.*, 2017). However, we could not identify any homologs to the essential pilin-associated c-type cytochrome OmcS (Shrestha *et al.*, 2013; Holmes *et al.*, 2017). We found an ortholog to a novel metal reduction complex in *Desulfotomaculum reducens* (Dred_1685–1686) (Otwell *et al.*, 2015) in SbA2 (SBA2_100009–10). This complex was shown to reduce Fe(III), Cr(VI), and U(VI) with NADH as the electron donor, but its physiological role is unresolved (Otwell *et al.*, 2015).

Import and phosphorylation of glucose

All genomes except SbA7 harbour at least one cytoplasmic glucokinase (*glk/glcK*) (Supplementary Table S2m). Cytoplasmic glucokinases are required to utilize glucose released by cytoplasmic polysaccharides degradation, but are not required for growth on glucose, as extracellular glucose can be imported and phosphorylated by the phosphotransferase system (PTS). The PTS is missing in all MAGs – Enzyme I and histidine protein are both not present. Only one fragment of a mannitol-specific enzyme IIBC component is found in SbA2 (SBA2_130007). Alternately, Lindner *et al.* (2011) demonstrated that inositol permeases IolT1/IolT2 are low-affinity glucose permeases, and together with glucokinases can replace the PTS in Corynebacterium glutamicum. *iolT1* and *iolT2* match TIGRFAM's sugar porter motif TIGR00879, which we also find in genes in all genomes but SbA2 (data not shown). These could putatively transport glucose, but also other sugars or inositol.

N-acetylgalactosamine degradation

N-acetylgalactosamine (GalNAc) degradation consists of five steps before entering glycolysis: (1) N -acetylgalactosamine kinase, (2) N-acetylgalactosamine-6-phosphate deacetylase, (3) galactosamine-6-phosphate deaminase, (4) tagatose-6-phosphate kinase, and (5) tagatose-bisphosphate aldolase (Figure 3, Supplementary Table S2l). Neither GalNAc-specific PTS genes or N-acetylgalactosamine kinase (*aga*K) were identified. However, other sugar kinases of are present, e.g., *glc*K-type glucokinases. Not all sugar kinases are specific for only one substrate (e.g., Reith *et al.*, 2011), therefore some of the those could putatively act as N-acetylgalactosamine kinases. N-acetylglucosamine-6 -phosphate deacetylase (*nag*A) was found in six MAGs. *E. coli* possesses two homologous N -acetylglucosamine-6-phosphate deacetylases (*nag*A and *aga*A, COG1820), both of whom can utilize GalNAc and N-acetylglucosamine (GlcNAc) (Hu *et al.*, 2013). Thereby it is likely the identified genes code for bifunctional deacetylases as well. Galactosamine-6-phosphate deaminase (AgaS), found in SbA1, SbA7, and SbA6, converts D-galactosamine 6-phosphate to D-tagatofuranose 6-phosphate. PfkB, found in SbA3, SbA4, and SbA2 is a bifunctional 6-phosphofructokinase and tagatose-6-phosphate kinase in *E. coli* (Babul, 1978). Most MAGs contain the last enzyme needed, tagatose-bisphosphate aldolase (LacD), which is a class I aldolase that produces glycerone phosphate and D-glyceraldehyde 3-phosphate. The *E. coli* class II aldolases of the same function (*kba*YZ, *gat*YZ) are heteromeric. Only the noncatalytic subunit *kbaZ* is found in SbA1 (SBA1_1710004). *kba*Y was never found.

Lactate, propionate, and butyrate metabolism

Six of the acidobacterial MAGs harbour four different types of L-lactate dehydrogenases, while SbA6 has none (Supplementary Table S2o). NAD-dependent L-lactate dehydrogenase Ldh (SbA7 and SbA5) ferments pyruvate to L-lactate anaerobically, while the FMN-dependent L-lactate dehydrogenase LldD (SbA1, SbA7, SbA3, and SbA4), L-lactate/D-lactate/glycolate dehydrogenase GlcDEF (SbA5, SbA3,

and SbA4) and, LUD-type L-lactate dehydrogenase LutABC (SbA5, SbA2, SbA3, and SbA4) utilize L-lactate as an energy and carbon source. We found putative D-lactate dehydrogenases (Dld) (SbA1, SbA5, and SbA2), which probably convert D-lactate to pyruvate. These are homologs to *Archaeoglobus* fulgidus Dld (AF_0394) and mitochondrial D-lactate dehydrogenases (<30% identity).

With the exception of SbA2, all MAGs contain key genes for propionate oxidation with complete pathways found in SbA1 and SbA5 (Supplementary Table S2o). Conversion of propionate to propionyl-CoA is performed by a CoA transferase. Propionyl-CoA:succinate CoA transferase ScpC is encoded in SbA1 and acetate CoA-transferase YdiF, a family 1 CoA transferase, which has propionyl-CoA:acetate CoA transferase activity (Rangarajan *et al.*, 2005), is encoded in SbA5 and SbA4 (Supplementary Table S2o). Other family 1 CoA transferase genes (IPR004165) are present,except in SbA2. It is unclear if these can utilize propionate as well, however it was proposed before for *Desulfotomaculum kuznetsovii* (Visser *et al.*, 2013). The main subunit gene of the propionyl-CoA carboxylase (PccB), which produces (*S*)-methylmalonyl-CoA, is present in all six MAGs. The propionyl-CoA carboxylase biotin carboxylase subunit and biotin carboxyl carrier protein are, however, missing in SbA7 and SbA4. Stereochemical inversion to (*R*)-methylmalonyl-CoA is performed by methylmalonyl-CoA epimerase (Mce), which is encoded in the same MAGs as ScpC/YdiF. Methylmalonyl-CoA mutase, catalyzing the final step in the pathway, is present in all six MAGs (Supplementary Table S2o).

Various putative beta-oxidation genes are present in all MAGs, however the substrate specificities of their encoded enzymes are unclear. For the case of butyrate oxidation, the physiological mechanisms are resolved in detail in the syntrophic organism *Syntrophomonas wolfei* (Schmidt *et al.*, 2013). No orthologs to the key enzyme butyryl-CoA dehydrogenase (Swol_1933/Swol_2052) are present in any of the MAGs, therefore it is unlikely they can perform (syntrophic) butyrate oxidation.

Differential gene expression between anoxic microcosm incubations

To analyze changes in gene expression of SbA1–7 during the anoxic peat soil incubations, we performed pairwise comparisons between different treatments and time points: (1) at every time point and for each added substrate we compared the microcosms amended with sulfate to those without external sulfate (i.e., stimulation or downregulation caused by sulfate), (2) at every time point and separately for sulfate-stimulated incubations and no-sulfate-controls we compared microcosms amended with substrate to the no-substrate-controls (i.e., up- or downregulation caused by formate, acetate, propionate, lactate, or butyrate); and (3) for each treatment we compared the early time point (8 days) to the late time point (36 days) (Supplementary Table S3a). When compared to the gene expression changes between the native soil and the incubations, less genes were upregulated between different incubations treatments. Differentially expressed genes included dissimilatory sulfur genes, hydrogen metabolism genes, electron transfer genes, and a few genes belonging to the tricarboxylic acid cycle (Supplementary Table S3a, Supplementary Figure S7).

Compared to the butyrate-only incubation, expression of *sat*, *apr*BA, *qmo*BC, *dsr*AB, *dsr*N, *dsrT*, and *dsrL* of SbA2 was induced upon addition of sulfate and butyrate. One subunit of (2*R*)-sulfolactate sulfo-lyase (*suyB*) was overexpressed in SbA4 in incubations with sulfate and formate. Compared to the no-substrate-controls, we observed significant overexpression of some sulfur metabolism genes from SbA2, SbA3, and SbA7 in formate-, propionate-, lactate-, and/or butyrate-amended incubations, with and/or without supplemental sulfate (Supplementary Table S3a, Supplementary Figure S7). However, we observed no significant expression changes in the genes that are possibly involved in oxidation of the amended substrates. Moderate expression of sulfate reduction genes without addition of external sulfate is expected due to cryptic sulfur cycling under anoxic conditions (Pester *et al.*, 2012a). The peat soil microcosms without external sulfate contained low amounts of endogenous sulfate (24±6 µM) that was only depleted after 11–25 days of incubation (Hausmann *et al.*, 2016).

Group 3 hydrogenase gene were affected by substrate amendment in incubation with and/or without external sulfate added. Group 3b hydrogenase (*hyhBCSL*) of SbA2 was stimulated by all substrates except acetate. Group 3c hydrogenase (*mvhDCA*) of SbA4 was significantly downregulated after the addition of propionate, lactate, and butyrate. Group 3d hydrogenase (*hoxEFYH*) of SbA1 was significantly overexpressed in butyrate-amended microcosms. HoxF of SbA2 was overexpressed when lactate or butyrate was added. Group 3 hydrogenases are cytoplasmic and possibly bidirectional (Greening *et al.*, 2016), leaving it unresolved if hydrogen is produced from the substrates or if hydrogen is provided from a substrate-utilizing syntrophic partner.

Supplementary References

Babul J. (1978). Phosphofructokinases from *Escherichia coli*: purification and characterization of the nonallosteric isozyme. *J Biol Chem* **253**: 4350–4355.

Buggy J, Bauer CE. (1995). Cloning and characterization of *senC*, a gene involved in both aerobic respiration and photosynthesis gene expression in *Rhodobacter capsulatus*. *J Bacteriol* **177**: 6958–6965.

Bühler D, Rossmann R, Landolt S, Balsiger S, Fischer H-M, Hennecke H. (2010). Disparate pathways for the biogenesis of cytochrome oxidases in *Bradyrhizobium japonicum*. *J Biol Chem* **285**: 15704–15713.

Cole JR, Wang Q, Fish JA, Chai B, McGarrell DM, Sun Y *et al.* (2014). Ribosomal Database Project: data and tools for high throughput rRNA analysis. *Nucleic Acids Res* **42**: D633–D642.

Cook GM, Poole RK. (2016). A bacterial oxidase like no other? *Science* **352**: 518–519.

Coursolle D, Gralnick JA. (2010). Modularity of the Mtr respiratory pathway of *Shewanella oneidensis* strain MR-1. *Mol Microbiol* **77**: 995–1008.

Dahl C, Friedrich CG. (2008). Microbial Sulfur Metabolism. Springer, Berlin Heidelberg.

Daims H, Lücker S, Wagner M. (2016). A new perspective on microbes formerly known as nitrite-oxidizing bacteria. *Trends Microbiol* **24**: 699–712.

Dannenberg S, Kroder M, Dilling W, Cypionka H. (1992). Oxidation of H_2, organic compounds and inorganic sulfur compounds coupled to reduction of O_2 or nitrate by sulfate-reducing bacteria. *Arch Microbiol* **158**: 93–99.

Dibrova DV, Galperin MY, Mulkidjanian AY. (2010). Characterization of the N-ATPase, a distinct, laterally transferred Na^+-translocating form of the bacterial F-type membrane ATPase. *Bioinformatics* **26**: 1473–1476.

Dunn SD, Kellner E, Lill H. (2001). Specific heterodimer formation by the cytoplasmic domains of the *b* and *b′* subunits of cyanobacterial ATP synthase. *Biochemistry* **40**: 187–192.

Eisen JA, Nelson KE, Paulsen IT, Heidelberg JF, Wu M, Dodson RJ *et al.* (2002). The complete genome sequence of *Chlorobium tepidum* TLS, a photosynthetic, anaerobic, green-sulfur bacterium. *Proc Natl Acad Sci USA* **99**: 9509–9514.

Friedrich T, Dekovic DK, Burschel S. (2016). Assembly of the *Escherichia coli* NADH:ubiquinone oxidoreductase (respiratory complex I). *Biochim Biophys Acta* **1857**: 214–223.

Gaby JC, Buckley DH. (2014). A comprehensive aligned *nifH* gene database: a multipurpose tool for

studies of nitrogen-fixing bacteria. *Database (Oxford)* **2014**: bau001.

Garcia Costas AM, Liu Z, Tomsho LP, Schuster SC, Ward DM, Bryant DA. (2012). Complete genome of *Candidatus* Chloracidobacterium thermophilum, a chlorophyll-based photoheterotroph belonging to the phylum *Acidobacteria*. *Environ Microbiol* **14**: 177–190.

Ghosh W, Dam B. (2009). Biochemistry and molecular biology of lithotrophic sulfur oxidation by taxonomically and ecologically diverse bacteria and archaea. *FEMS Microbiol Rev* **33**: 999–1043.

Giuffrè A, Borisov VB, Arese M, Sarti P, Forte E. (2014). Cytochrome *bd* oxidase and bacterial tolerance to oxidative and nitrosative stress. *Biochim Biophys Acta* **1837**: 1178–1187.

Greening C, Biswas A, Carere CR, Jackson CJ, Taylor MC, Stott MB *et al.* (2016). Genomic and metagenomic surveys of hydrogenase distribution indicate H_2 is a widely utilised energy source for microbial growth and survival. *ISME J* **10**: 761–777.

Guest JR. (1981). Partial replacement of succinate dehydrogenase function by phage- and plasmid-specified fumarate reductase in *Escherichia coli*. *J Gen Microbiol* **122**: 171–179.

Hausmann B, Knorr K-H, Schreck K, Tringe SG, Glavina del Rio T, Loy A *et al.* (2016). Consortia of low-abundance bacteria drive sulfate reduction-dependent degradation of fermentation products in peat soil microcosms. *The ISME Journal* **10**: 2365–2375.

Holmes DE, Shrestha PM, Walker DJF, Dang Y, Nevin KP, Woodard TL *et al.* (2017). Metatranscriptomic evidence for direct interspecies electron transfer between *Geobacter* and *Methanothrix* species in methanogenic rice paddy soils. *Appl Environ Microbiol* **83**: e00223–17.

Hu Z, Patel IR, Mukherjee A. (2013). Genetic analysis of the roles of *aga*A, *aga*I, and *aga*S genes in the N-acetyl-D-galactosamine and D-galactosamine catabolic pathways in *Escherichia coli* strains O157:H7 and C. *BMC Microbiol* **13**: 94.

Huang CJ, Barrett EL. (1991). Sequence analysis and expression of the *Salmonella typhimurium asr* operon encoding production of hydrogen sulfide from sulfite. *J Bacteriol* **173**: 1544–1553.

Huerta-Cepas J, Szklarczyk D, Forslund K, Cook H, Heller D, Walter MC *et al.* (2016). eggNOG 4.5: a hierarchical orthology framework with improved functional annotations for eukaryotic, prokaryotic and viral sequences. *Nucleic Acids Res* **44**: D286–D293.

Hyatt D, Chen G-L, Locascio PF, Land ML, Larimer FW, Hauser LJ. (2010). Prodigal: prokaryotic gene recognition and translation initiation site identification. *BMC Bioinformatics* **11**: 119.

Iguchi H, Yurimoto H, Sakai Y. (2010). Soluble and particulate methane monooxygenase gene clusters of the type I methanotroph *Methylovulum miyakonense* HT12. *FEMS Microbiol Lett* **312**: 71–76.

Johnson EF, Mukhopadhyay B. (2005). A new type of sulfite reductase, a novel coenzyme F_{420} -dependent enzyme, from the methanarchaeon *Methanocaldococcus jannaschii*. *J Biol Chem* **280**: 38776–38786.

Jones P, Binns D, Chang H-Y, Fraser M, Li W, McAnulla C *et al.* (2014). InterProScan 5: genome-scale protein function classification. *Bioinformatics* **30**: 1236–1240.

Katoh K, Standley DM. (2013). MAFFT multiple sequence alignment software version 7: improvements in performance and usability. *Mol Biol Evol* **30**: 772–780.

Klenk HP, Clayton RA, Tomb JF, White O, Nelson KE, Ketchum KA *et al.* (1997). The complete genome sequence of the hyperthermophilic, sulphate-reducing archaeon *Archaeoglobus fulgidus*. *Nature* **390**: 364–370.

Kraft B, Strous M, Tegetmeyer HE. (2011). Microbial nitrate respiration–genes, enzymes and environmental distribution. *J Biotechnol* **155**: 104–117.

Krogh A, Larsson B, von Heijne G, Sonnhammer ELL. (2001). Predicting transmembrane protein topology with a hidden Markov model: application to complete genomes. *J Mol Biol* **305**: 567–580.

Kuever J, Visser M, Loeffler C, Boll M, Worm P, Sousa DZ *et al.* (2014). Genome analysis of *Desulfotomaculum gibsoniae* strain Groll^{T} a highly versatile Gram-positive sulfate-reducing bacterium. *Stand Genomic Sci* **9**: 821–839.

Langmead B, Salzberg SL. (2012). Fast gapped-read alignment with Bowtie 2. *Nat Methods* **9**: 357–359.

Lartillot N, Lepage T, Blanquart S. (2009). PhyloBayes 3: a Bayesian software package for phylogenetic reconstruction and molecular dating. *Bioinformatics* **25**: 2286–2288.

Laska S, Lottspeich F, Kletzin A. (2003). Membrane-bound hydrogenase and sulfur reductase of the hyperthermophilic and acidophilic archaeon *Acidianus ambivalens*. *Microbiology* **149**: 2357–2371.

Liao Y, Smyth GK, Shi W. (2014). featureCounts: an efficient general purpose program for assigning sequence reads to genomic features. *Bioinformatics* **30**: 923–930.

Lindner SN, Seibold GM, Henrich A, Krämer R, Wendisch VF. (2011). Phosphotransferase system-independent glucose utilization in *Corynebacterium glutamicum* by inositol permeases and glucokinases. *Appl Environ Microbiol* **77**: 3571–3581.

Lombard V, Golaconda Ramulu H, Drula E, Coutinho PM, Henrissat B. (2014). The carbohydrate-active enzymes database (CAZy) in 2013. *Nucleic Acids Res* **42**: D490–D495.

Love MI, Huber W, Anders S. (2014). Moderated estimation of fold change and dispersion for RNA-seq data with DESeq2. *Genome Biol* **15**: 550.

Maklashina E, Berthold DA, Cecchini G. (1998). Anaerobic expression of *Escherichia coli* succinate dehydrogenase: functional replacement of fumarate reductase in the respiratory chain during anaerobic growth. *J Bacteriol* **180**: 5989–5996.

Mardanov AV, Panova IA, Beletsky AV, Avakyan MR, Kadnikov VV, Antsiferov DV *et al.* (2016). Genomic insights into a new acidophilic, copper-resistant *Desulfosporosinus* isolate from the oxidized tailings area of an abandoned gold mine Lueders T (ed). *FEMS Microbiol Ecol* **92**: fiw111.

Marietou A. (2016). Nitrate reduction in sulfate-reducing bacteria. *FEMS Microbiol Lett* **363**: fnw155.

Marzocchi U, Trojan D, Larsen S, Meyer RL, Revsbech NP, Schramm A *et al.* (2014). Electric coupling between distant nitrate reduction and sulfide oxidation in marine sediment. *ISME J* **8**: 1682–1690.

Männistö MK, Rawat S, Starovoytov V, Häggblom MM. (2012). *Granulicella arctica* sp. nov., *Granulicella mallensis* sp. nov., *Granulicella tundricola* sp. nov. and *Granulicella sapmiensis* sp. nov., novel acidobacteria from tundra soil. *Int J Syst Evol Microbiol* **62**: 2097–2106.

Mitchell A, Chang H-Y, Daugherty L, Fraser M, Hunter S, Lopez R *et al.* (2015). The InterPro protein

families database: the classification resource after 15 years. *Nucleic Acids Res* **43**: D213–D221.

Mizuno N, Voordouw G, Miki K, Sarai A, Higuchi Y. (2003). Crystal structure of dissimilatory sulfite reductase D (DsrD) protein–possible interaction with B- and Z-DNA by its winged-helix motif. *Structure* **11**: 1133–1140.

Moretti S, Armougom F, Wallace IM, Higgins DG, Jongeneel CV, Notredame C. (2007). The M-Coffee web server: a meta-method for computing multiple sequence alignments by combining alternative alignment methods. *Nucleic Acids Res* **35**: W645–W648.

Morris RL, Schmidt TM. (2013). Shallow breathing: bacterial life at low O_2. *Nat Rev Microbiol* **11**: 205–212.

Müller AL, Kjeldsen KU, Rattei T, Pester M, Loy A. (2015). Phylogenetic and environmental diversity of DsrAB-type dissimilatory (bi)sulfite reductases. *ISME J* **9**: 1152–1165.

Nguyen L-T, Schmidt HA, Haeseler A von, Minh BQ. (2015). IQ-TREE: a fast and effective stochastic algorithm for estimating maximum-likelihood phylogenies. *Mol Biol Evol* **32**: 268–274.

Otwell AE, Sherwood RW, Zhang S, Nelson OD, Li Z, Lin H *et al.* (2015). Identification of proteins capable of metal reduction from the proteome of the Gram-positive bacterium *Desulfotomaculum reducens* MI-1 using an NADH-based activity assay. *Environ Microbiol* **17**: 1977–1990.

Parks DH, Imelfort M, Skennerton CT, Hugenholtz P, Tyson GW. (2015). CheckM: assessing the quality of microbial genomes recovered from isolates, single cells, and metagenomes. *Genome Res* **25**: 1043–1055.

Pereira IAC, Ramos AR, Grein F, Marques MC, Marques da Silva S, Venceslau SS. (2011). A comparative genomic analysis of energy metabolism in sulfate reducing bacteria and archaea. *Front Microbiol* **2**: 69.

Pereira MM, Santana M, Teixeira M. (2001). A novel scenario for the evolution of haem-copper oxygen reductases. *Biochim Biophys Acta* **1505**: 185–208.

Pester M, Bittner N, Deevong P, Wagner M, Loy A. (2010). A 'rare biosphere' microorganism contributes to sulfate reduction in a peatland. *ISME J* **4**: 1591–1602.

Pester M, Knorr K-H, Friedrich MW, Wagner M, Loy A. (2012a). Sulfate-reducing microorganisms in wetlands – fameless actors in carbon cycling and climate change. *Front Microbiol* **3**: 72.

Pester M, Maixner F, Berry D, Rattei T, Koch H, Lücker S *et al.* (2014). *NxrB* encoding the beta subunit of nitrite oxidoreductase as functional and phylogenetic marker for nitrite-oxidizing *Nitrospira*. *Environ Microbiol* **16**: 3055–3071.

Pester M, Rattei T, Flechl S, Gröngröft A, Richter A, Overmann J *et al.* (2012b). *amo*A-based consensus phylogeny of ammonia-oxidizing archaea and deep sequencing of *amo*A genes from soils of four different geographic regions. *Environ Microbiol* **14**: 525–539.

Plugge CM, Henstra AM, Worm P, Swarts DC, Paulitsch-Fuchs AH, Scholten JCM *et al.* (2012). Complete genome sequence of *Syntrophobacter fumaroxidans* strain ($MPOB^T$). *Stand Genomic Sci* **7**: 91–106.

Price MN, Dehal PS, Arkin AP. (2010). FastTree 2 – approximately maximum-likelihood trees for large alignments. *PLoS One* **5**: e9490.

Quast C, Pruesse E, Yilmaz P, Gerken J, Schweer T, Yarza P *et al.* (2013). The SILVA ribosomal RNA gene database project: improved data processing and web-based tools. *Nucleic Acids Res* **41**: D590–D596.

R Core Team. (2017). R: a language and environment for statistical computing. R Foundation for Statistical Computing: Vienna, Austria. http://www.r-project.org/.

Rabus R, Hansen TA, Widdel F. (2013). Dissimilatory sulfate- and sulfur-reducing prokaryotes. In: Rosenberg E, DeLong EF, Lory S, Stackebrandt E, Thompson F (eds). *The Prokaryotes – prokaryotic physiology and biochemistry*. Springer Berlin Heidelberg, pp 309–404.

Rabus R, Ruepp A, Frickey T, Rattei T, Fartmann B, Stark M *et al.* (2004). The genome of *Desulfotalea psychrophila*, a sulfate-reducing bacterium from permanently cold Arctic sediments. *Environ Microbiol* **6**: 887–902.

Ramel F, Amrani A, Pieulle L, Lamrabet O, Voordouw G, Seddiki N *et al.* (2013). Membrane-bound oxygen reductases of the anaerobic sulfate-reducing *Desulfovibrio vulgaris* Hildenborough: roles in oxygen defence and electron link with periplasmic hydrogen oxidation. *Microbiology* **159**: 2663–2673.

Rangarajan ES, Li Y, Ajamian E, Iannuzzi P, Kernaghan SD, Fraser ME *et al.* (2005). Crystallographic trapping of the glutamyl-CoA thioester intermediate of family I CoA transferases. *J Biol Chem* **280**: 42919–42928.

Refojo PN, Teixeira M, Pereira MM. (2012). The Alternative complex III: properties and possible mechanisms for electron transfer and energy conservation. *Biochim Biophys Acta* **1817**: 1852–1859.

Reith J, Berking A, Mayer C. (2011). Characterization of an N-acetylmuramic acid/N-acetylglucosamine kinase of *Clostridium acetobutylicum*. *J Bacteriol* **193**: 5386–5392.

Schmidt A, Müller N, Schink B, Schleheck D. (2013). A proteomic view at the biochemistry of syntrophic butyrate oxidation in *Syntrophomonas wolfei*. *PLoS One* **8**: e56905.

Shi L, Chen B, Wang Z, Elias DA, Mayer MU, Gorby YA *et al.* (2006). Isolation of a high-affinity functional protein complex between OmcA and MtrC: two outer membrane decaheme *c*-type cytochromes of *Shewanella oneidensis* MR-1. *J Bacteriol* **188**: 4705–4714.

Shrestha PM, Rotaru A-E, Summers ZM, Shrestha M, Liu F, Lovley DR. (2013). Transcriptomic and genetic analysis of direct interspecies electron transfer. *Appl Environ Microbiol* **79**: 2397–2404.

Simon J, Kroneck PMH. (2013). Microbial sulfite respiration.

Strittmatter AW, Liesegang H, Rabus R, Decker I, Amann J, Andres S *et al.* (2009). Genome sequence of *Desulfobacterium autotrophicum* HRM2, a marine sulfate reducer oxidizing organic carbon completely to carbon dioxide. *Environ Microbiol* **11**: 1038–1055.

Sumi M, Yohda M, Koga Y, Yoshida M. (1997). F_0F_1-ATPase genes from an archaebacterium, *Methanosarcina barkeri*. *Biochem Biophys Res Commun* **241**: 427–433.

The UniProt Consortium. (2015). UniProt: a hub for protein information. *Nucleic Acids Res* **43**: D204–D212.

Thorup C, Schramm A, Findlay AJ, Finster KW, Schreiber L. (2017). Disguised as a sulfate reducer: growth of the deltaproteobacterium *Desulfurivibrio alkaliphilus* by sulfide oxidation with nitrate. *MBio* **8**: e00671–17.

Vallenet D, Calteau A, Cruveiller S, Gachet M, Lajus A, Josso A *et al.* (2017). MicroScope in 2017: an expanding and evolving integrated resource for community expertise of microbial genomes. *Nucleic Acids Res* **45**: D517–D528.

Varghese NJ, Mukherjee S, Ivanova N, Konstantinidis KT, Mavrommatis K, Kyrpides NC *et al.* (2015). Microbial species delineation using whole genome sequences. *Nucleic Acids Res* **43**: 6761–6771.

Visser M, Worm P, Muyzer G, Pereira I a C, Schaap PJ, Plugge CM *et al.* (2013). Genome analysis of *Desulfotomaculum kuznetsovii* strain 17^T reveals a physiological similarity with *Pelotomaculum thermopropionicum* strain SI^T. *Stand Genomic Sci* **8**: 69–87.

Ward NL, Challacombe JF, Janssen PH, Henrissat B, Coutinho PM, Wu M *et al.* (2009). Three genomes from the phylum *Acidobacteria* provide insight into the lifestyles of these microorganisms in soils. *Appl Environ Microbiol* **75**: 2046–2056.

Wasmund K, Mußmann M, Loy A. (2017). The life sulfuric: microbial ecology of sulfur cycling in marine sediments. *Environ Microbiol Rep* **9**: 323–344.

Watanabe T, Kojima H, Fukui M. (2016). Identity of major sulfur-cycle prokaryotes in freshwater lake ecosystems revealed by a comprehensive phylogenetic study of the dissimilatory adenylylsulfate reductase. *Sci Rep* **6**: 36262.

Weber KA, Achenbach LA, Coates JD. (2006). Microorganisms pumping iron: anaerobic microbial iron oxidation and reduction. *Nat Rev Microbiol* **4**: 752–764.

Weissgerber T, Zigann R, Bruce D, Chang Y-j, Detter JC, Han C *et al.* (2011). Complete genome sequence of *Allochromatium vinosum* DSM 180^T. *Stand Genomic Sci* **5**: 311–330.

Wu AJ, Penner-Hahn JE, Pecoraro VL. (2004). Structural, spectroscopic, and reactivity models for the manganese catalases. *Chem Rev* **104**: 903–938.

Yin Y, Mao X, Yang J, Chen X, Mao F, Xu Y. (2012). dbCAN: a web resource for automated carbohydrate-active enzyme annotation. *Nucleic Acids Res* **40**: W445–W451.

Supplementary Tables

Supplementary Table S1

Taxonomy, genome characteristics, and abundance measures of the DsrAB-encoding MAGs. Estimation of completeness and contamination was performed with checkM. Genome abundance estimates are the fraction of metagenomic reads mapped to each MAG in relation to all quality filtered reads. Values given for native soil are averages of both native soil metagenomes. mRNA abundance estimates (only acidobacterial MAGs) are the fraction of fragments (paired-end reads) mapped to all of each MAG's CDS in relation to all quality filtered fragments. Standard deviation of three replicates is given. Fraction of expressed CDS in native soil (%) is given only for acidobacterial MAGs.

Supplementary Table S2

Curated annotation tables of SbA1–7: (a) dissimilatory sulfur metabolism; (b–h) respiratory complexes I–V: NADH dehydrogenases (NDH, b), succinate dehydrogenase (SDH, c), quinol–cytochrome-c reductases (CIII/ACIII, d/e), low- (LATO, f) and high-affinity (HATO, g) terminal oxidases, and ATP synthases (h); (i) stress (superoxide detoxification), (j) formate dehydrogenases (FDH), hydrogenases (Hase); (k) cytoplasmic electron transport systems; (l) N-acetylgalactosamine degradation; (m) glycolysis (and gluconeogenesis), pentose phosphate pathway, and Entner-Doudoroff pathway; (n) citric acid cycle (TCA); (o) pyruvate, acetate, propionate, and related metabolisms; (p) dissimilatory metal metabolism. Columns provide functional categories (only a, m, o), pathway step number and/or proposed direction (only m, o), product (enzyme, transporter) names with EC and TC numbers where appropriate, subunit names or descriptions where appropriate, gene names, and locus numbers per MAG. Products with multiple copies per MAG are separated into more than one column (only b–f, j). Fragmented genes (assembly or biological artefacts) are marked with downward arrows (↓) after their loci numbers. TM, transmembrane subunit. [1] or [2] indicates the first or last CDS on a scaffold (depending on the reading frame).

The following metabolic marker genes are absent in SbA1–7 MAGs: Inorganic sulfur metabolism (Wasmund *et al.*, 2017): *sox*ABXYZ (Sox enzyme machinery), *tsd*A, thiosulfate dehydrogenase; *otr*, octaheme tetrathionate reductase; *phs*ABC, thiosulfate reductase; *psr*ABC, polysulfide reductase; *sre*ABC, sulfur reductase (Laska *et al.*, 2003); *asr*ABC, trimeric dissimilatory sulfite reductase (anaerobic sulfite reductase) (Huang and Barrett, 1991); *fsr*, coenzyme F_{420}-dependent sulfite reductase (Johnson and Mukhopadhyay, 2005). ATPases: *atp*DCQRBEFAG, N-ATPase (alternative “archaeal-type” F_oF_1-ATPase) (Sumi *et al.*, 1997; Dibrova *et al.*, 2010). Dissimilatory nitrate reduction to ammonium (DNRA) (Kraft *et al.*, 2011): *nap*AB, periplasmic nitrate reductase (NAP), catalytic subunit is NapA; *nrf*A, periplasmic cytochrome c nitrite reductase (catalytic subunit). Denitrification (Kraft *et al.*, 2011): *nar*GHI, membrane bound cytoplasm-facing nitrate reductase (NAR), catalytic subunit is NarG; *nir*K or *nir*S, isofunctional but evolutionarily unrelated periplasmic nitric oxide-forming nitrite reductase (NIR); *nor*BC, membrane bound periplasm-facing nitric oxide reductase (NOR), catalytic subunit is NorB; *nos*Z, periplasmic nitrous oxide reductase (NOS). Nitrogen fixation: *nif*H, nitrogenase. Nitrification: *amo*CAB, ammonia monooxygenase (AMO); *nxr*AB, nitrite oxidoreductase (NXR). Methanotrophy (Iguchi *et al.*, 2010): *pmo*CAB, particulate methane monooxygenase (pMMO); *mmo*XYBZDC, soluble methane monooxygenase (sMMO). Photosynthesis: *psc*AB-*fmo*A, *Chloracidobacterium thermophilum* photosystem (Garcia Costas *et al.*, 2012); *psc*ABCD, *Chlorobium tepidum* photosystem (Eisen *et al.*, 2002); *puf*ABCMLH, *Allochromatium vinosum* photosystem (Weissgerber *et al.*, 2011). ROS defence: *kat*N, mono-functional, manganese catalase (EC 1.11.1.6) (Wu *et al.*, 2004); *kat*E/*kat*A, mono-functional, haem-containing catalase (EC 1.11.1.6). Hydrogenases: [FeFe] hydrogenases.

Supplementary Table S3

(a) Supplementary Table S2 deposited in machine-readable format including additional information. The length of CDS terminated by scaffold borders ([1] or [2] in strand column) is underestimated, as the true length is not known. bactNOG and NOG IDs were assigned by best-match principle. Ranks are based on averaged FPKM. Missing ranks indicate that expression was never detected in any replicate. Significant differential expression is shown separated by factor. Three-letter-codes are initials of amended substrates (F, A, P, L, B) or N for no-substrate-control, sulfate stimulation (S) or control without external sulfate (C), and early (E, 8 days) and late (L, 36 days) time points, followed by the $\log_2$ fold change. Over- or underexpression is indicated by arrows or smaller than/larger than signs. (b) Glycoside hydrolase genes identified with dbCAN.

Supplementary Table S4

Glycoside hydrolases genes summarized by EC numbers provided by the carbohydrate-active enzymes database. Note that glycoside hydrolases can have more than one EC number assigned and not every glycoside hydrolase family member may perform each of the enzymatic reactions.

Supplementary Figures

Supplementary Figure S1

Reductive bacterial-type DsrAB. Maximum likelihood tree was calculated by FastTree 2.1.9 (Price *et al.*, 2010) (LG model, 1000 resamplings) using a reference amino acid alignment with reductive bacterial-type DsrAB indel positions removed (Müller *et al.*, 2015) aligned with MAFFT 7.271 (Katoh and Standley, 2013). Branch supports equal to or greater than 0.9 are indicated by black circles. DsrAB sequences from MAGs and scaffolds are marked in bold. Binned acidobacterial DsrAB sequences are coloured analogous to Figure 3. Dashed branches represent incomplete *dsr*AB gene sequences (e.g., caused by a contig ending) that were sufficiently long to be included in the phylogenetic analysis (only *dsr*AB genes on scaffold 43ik were too short and omitted). The extent of subdivision 3 group is unclear and indicated by a dashed line. Outgroup sequences are shown in Supplementary Figure S2.

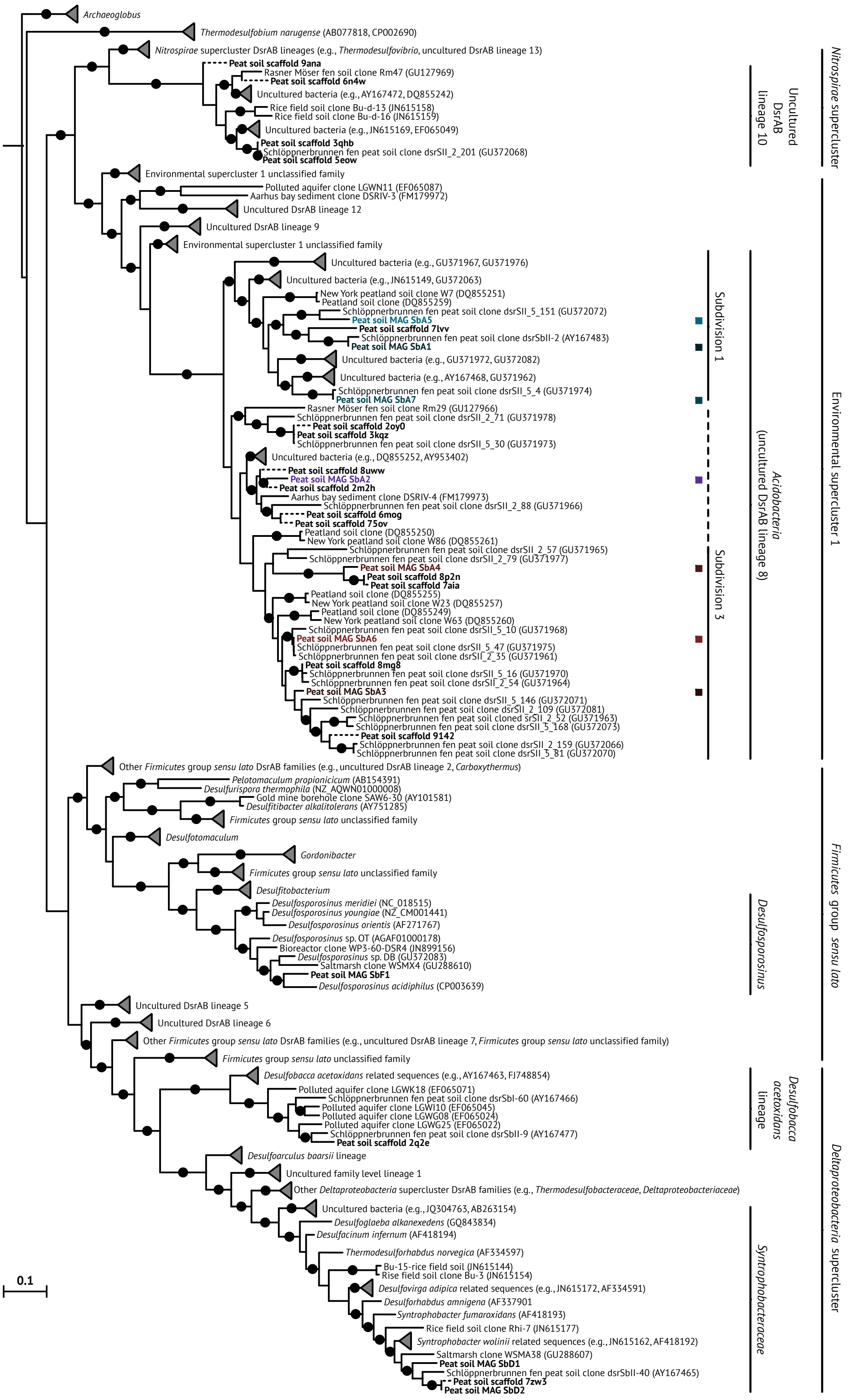
Archaeoglobus
Thermodesulfobium narugense (AB077818, CP002690)
Nitrospirae supercluster DsrAB lineages (e.g., Thermodesulfovibrio, uncultured DsrAB lineage 13)
Peat soil scaffold 9ana
Rasner Möser fen soil clone Rm47 (GU127969)
Peat soil scaffold 6n4w
Uncultured bacteria (e.g., AY167472, DQ855242)
Rice field soil clone Bu-d-13 (JN615158)
Rice field soil clone Bu-d-16 (JN615159)
Uncultured bacteria (e.g., JN615169, EF065049)
Peat soil scaffold 3qhb
Schlöppnerbrunnen fen peat soil clone dsrSII_2_201 (GU372068)
Peat soil scaffold 5eow
Environmental supercluster 1 unclassified family
Polluted aquifer clone LGWN11 (EF065087)
Aarhus bay sediment clone DSRIV-3 (FM179972)
Uncultured DsrAB lineage 12
Uncultured DsrAB lineage 9
Environmental supercluster 1 unclassified family
Uncultured bacteria (e.g., GU371967, GU371976)
Uncultured bacteria (e.g., JN615149, GU372063)
New York peatland soil clone W7 (DQ855251)
Peatland soil clone (DQ855259)
Schlöppnerbrunnen fen peat soil clone dsrSII_5_151 (GU372072)
Peat soil MAG SbA5
Peat soil scaffold 7lvv
Schlöppnerbrunnen fen peat soil clone dsrSbII-2 (AY167483)
Peat soil MAG SbA1
Uncultured bacteria (e.g., GU371972, GU372082)
Uncultured bacteria (e.g., AY167468, GU371962)
Schlöppnerbrunnen fen peat soil clone dsrSII_5_4 (GU371974)
Peat soil MAG SbA7
Rasner Möser fen soil clone Rm29 (GU127966)
Schlöppnerbrunnen fen peat soil clone dsrSII_2_71 (GU371978)
Peat soil scaffold 2oy0
Peat soil scaffold 3kqz
Schlöppnerbrunnen fen peat soil clone dsrSII_5_30 (GU371973)
Uncultured bacteria (e.g., DQ855252, AY953402)
Peat soil scaffold 8uww
Peat soil MAG SbA2
Peat soil scaffold 2m2h
Aarhus bay sediment clone DSRIV-4 (FM179973)
Schlöppnerbrunnen fen peat soil clone dsrSII_2_88 (GU371966)
Peat soil scaffold 6mog
Peat soil scaffold 75ov
Peatland soil clone (DQ855250)
New York peatland soil clone W86 (DQ855261)
Schlöppnerbrunnen fen peat soil clone dsrSII_2_57 (GU371965)
Schlöppnerbrunnen fen peat soil clone dsrSII_2_79 (GU371977)
Peat soil MAG SbA4
Peat soil scaffold 8p2n
Peat soil scaffold 7aia
Peatland soil clone (DQ855255)
New York peatland soil clone W23 (DQ855257)
Peatland soil clone (DQ855249)
New York peatland soil clone W63 (DQ855260)
Schlöppnerbrunnen fen peat soil clone dsrSII_5_10 (GU371968)
Peat soil MAG SbA6
Schlöppnerbrunnen fen peat soil clone dsrSII_5_47 (GU371975)
Schlöppnerbrunnen fen peat soil clone dsrSII_2_35 (GU371961)
Peat soil scaffold 8mg8
Schlöppnerbrunnen fen peat soil clone dsrSII_5_16 (GU371970)
Schlöppnerbrunnen fen peat soil clone dsrSII_2_54 (GU371964)
Peat soil MAG SbA3
Schlöppnerbrunnen fen peat soil clone dsrSII_5_146 (GU372071)
Schlöppnerbrunnen fen peat soil clone dsrSII_2_109 (GU372081)
Schlöppnerbrunnen fen peat soil cloned srSII_2_52 (GU371963)
Schlöppnerbrunnen fen peat soil clone dsrSII_5_168 (GU372073)
Peat soil scaffold 9142
Schlöppnerbrunnen fen peat soil clone dsrSII_2_159 (GU372066)
Schlöppnerbrunnen fen peat soil clone dsrSII_5_81 (GU372070)
Other Firmicutes group sensu lato DsrAB families (e.g., uncultured DsrAB lineage 2, Carboxythermus)
Pelotomaculum propionicicum (AB154391)
Desulfurispora thermophila (NZ_AQWN01000008)
Gold mine borehole clone SAW6-30 (AY101581)
Desulfitibacter alkalitolerans (AY751285)
Firmicutes group sensu lato unclassified family
Desulfotomaculum
Gordonibacter
Firmicutes group sensu lato unclassified family
Desulfitobacterium
Desulfosporosinus meridiei (NC_018515)
Desulfosporosinus youngiae (NZ_CM001441)
Desulfosporosinus orientis (AF271767)
Desulfosporosinus sp. OT (AGAF01000178)
Bioreactor clone WP3-60-DSR4 (JN899156)
Desulfosporosinus sp. DB (GU372083)
Saltmarsh clone WSMX4 (GU288610)
Peat soil MAG SbF1
Desulfosporosinus acidiphilus (CP003639)
Uncultured DsrAB lineage 5
Uncultured DsrAB lineage 6
Other Firmicutes group sensu lato DsrAB families (e.g., uncultured DsrAB lineage 7, Firmicutes group sensu lato unclassified family)
Firmicutes group sensu lato unclassified family
Desulfobacca acetoxidans related sequences (e.g., AY167463, FJ748854)
Polluted aquifer clone LGWK18 (EF065071)
Schlöppnerbrunnen fen peat soil clone dsrSbI-60 (AY167466)
Polluted aquifer clone LGWI10 (EF065045)
Polluted aquifer clone LGWG08 (EF065024)
Polluted aquifer clone LGWG25 (EF065022)
Schlöppnerbrunnen fen peat soil clone dsrSbII-9 (AY167477)
Peat soil scaffold 2q2e
Desulfoarculus baarsii lineage
Uncultured family level lineage 1
Other Deltaproteobacteria supercluster DsrAB families (e.g., Thermodesulfobacteraceae, Deltaproteobacteriaceae)
Uncultured bacteria (e.g., JQ304763, AB263154)
Desulfoglaeba alkanexedens (GQ843834)
Desulfacinum infernum (AF418194)
Thermodesulforhabdus norvegica (AF334597)
Bu-15-rice field soil (JN615144)
Rise field soil clone Bu-3 (JN615154)
Desulfovirga adipica related sequences (e.g., JN615172, AF334591)
Desulforhabdus amnigena (AF337901)
Syntrophobacter fumaroxidans (AF418193)
Rice field soil clone Rhi-7 (JN615177)
Syntrophobacter wolinii related sequences (e.g., JN615162, AF418192)
Saltmarsh clone WSMA38 (GU288607)
Peat soil MAG SbD1
Schlöppnerbrunnen fen peat soil clone dsrSbII-40 (AY167465)
Peat soil scaffold 7zw3
Peat soil MAG SbD2
0.1
Uncultured DsrAB lineage 10
Nitrospirae supercluster
Subdivision 1
Subdivision 3
Acidobacteria (uncultured DsrAB lineage 8)
Environmental supercluster 1
Desulfosporosinus
Firmicutes group sensu lato
Desulfobacca acetoxidans lineage
Syntrophobacteraceae
Deltaproteobacteria supercluster

Supplementary Figure S2

Oxidative bacterial-type DsrAB. Maximum likelihood phylogenetic tree was calculated by FastTree 2.1.9 (Price *et al.*, 2010) (LG model, 1000 resamplings) using a reference amino acid alignment with oxidative bacterial-type DsrAB indel positions removed (Müller *et al.*, 2015) aligned with MAFFT 7.271 (Katoh and Standley, 2013). Branch supports equal to or greater than 0.9 are indicated by black circles. DsrAB sequences from MAGs and scaffolds are marked in bold. Dashed branches represent partial DsrAB sequences on scaffolds.

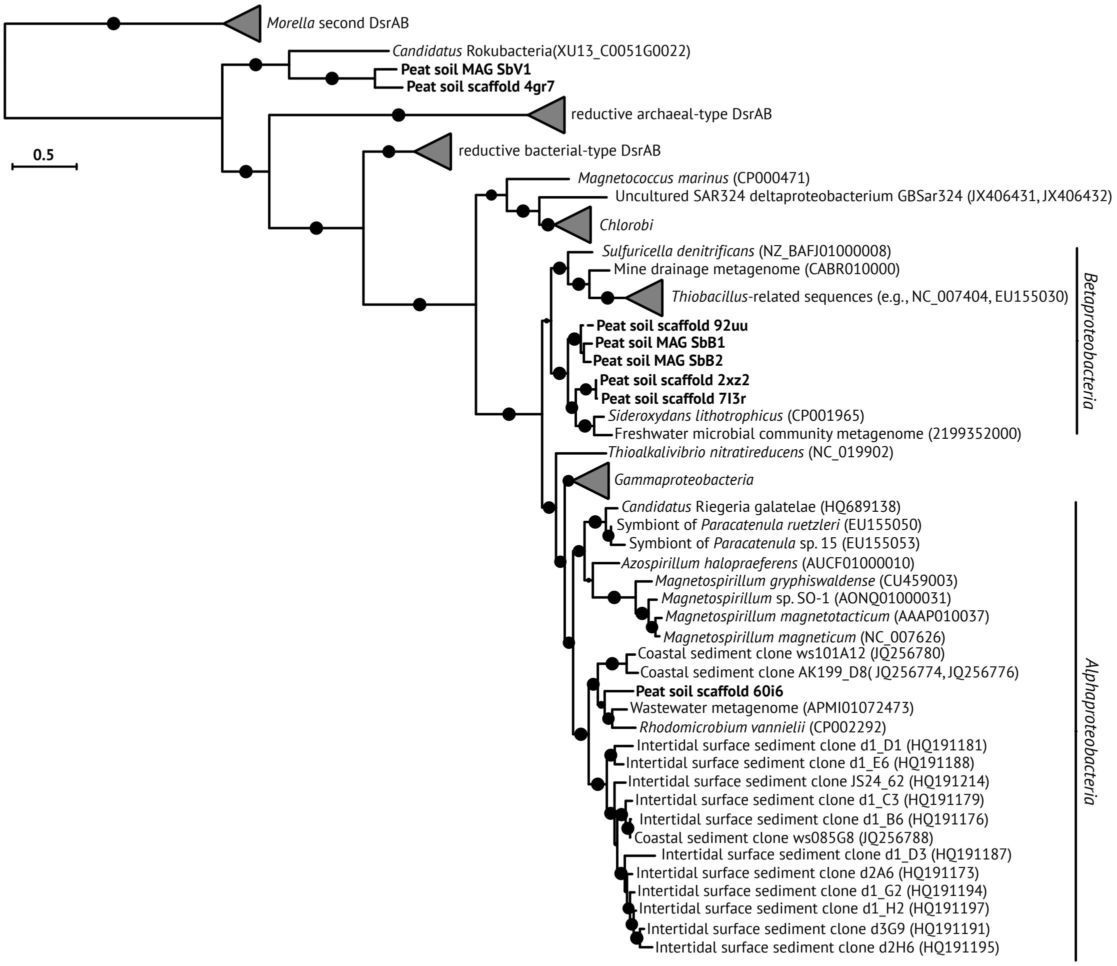

Supplementary Figure S3

Phylogenomic tree and pairwise average amino acid identities of *Acidobacteria* genomes and MAGs. NCBI assembly accessions are given in parentheses. Novel sequences from this study are marked in bold. *Acidobacterial* subdivisions are given below. Dendrograms are a phylogenomic tree calculated with phylobayes from a checkM-produced and -filtered amino acid alignment. All branches are supported >0.9. Genomes assemblies from the *Firmicutes*, *Proteobacteria*, and *Verrucomicrobia* were used as outgroup. MAG SbA2 has an AAI of 37–49% to the other genomes and MAGs but was not included in the figure because it lacks the marker genes used for the phylogenomic tree.

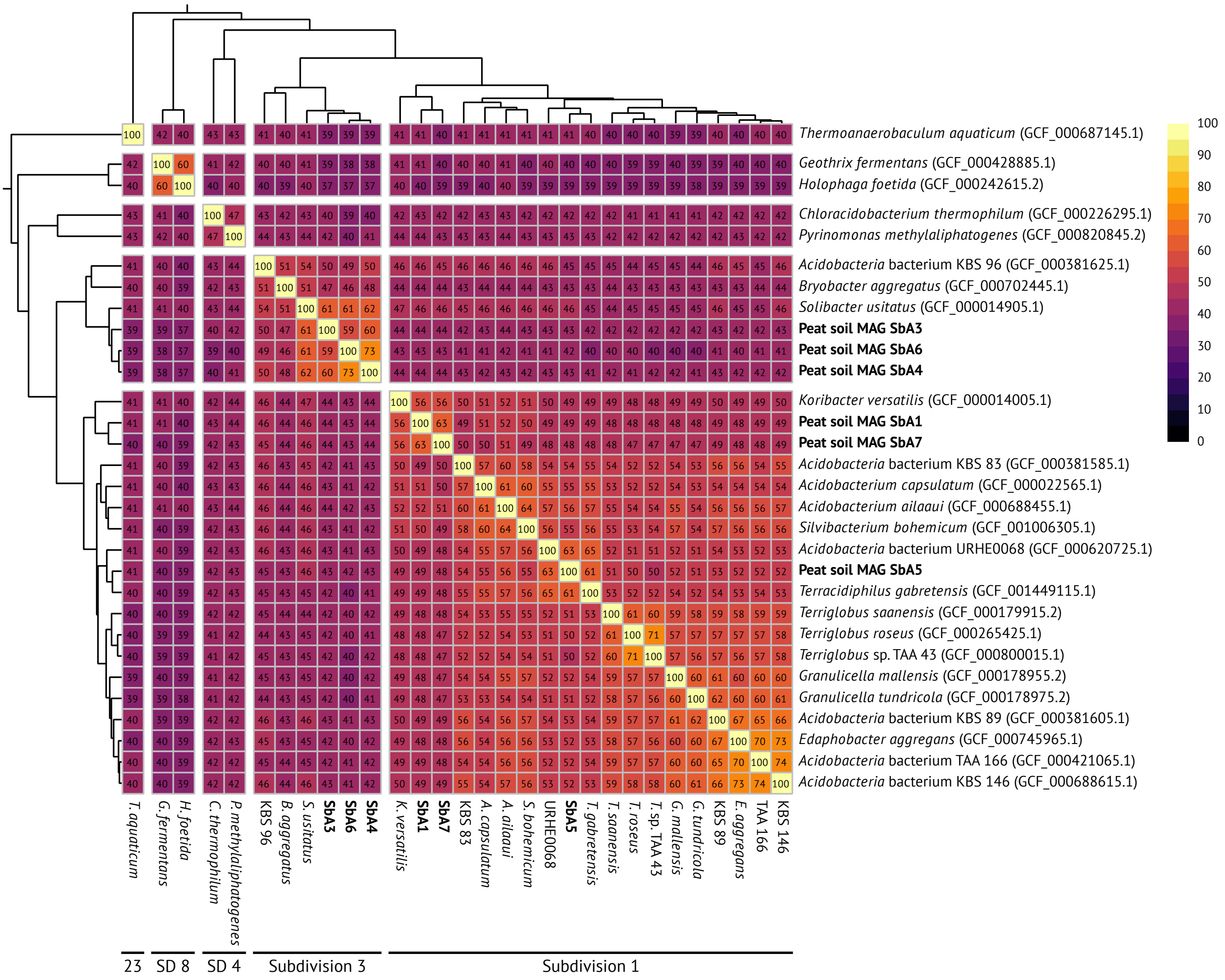

Supplementary Figure S4

AprBA phylogeny. AprBA sequences were collected from UniProt using the InterPro identifiers IPR011802 and IPR011803 for AprB and AprA, respectively. AprB and AprA sequences were reference-aligned to EggNOG's trimmed 08RS8 and 05CFK bactNOG alignments (Huerta-Cepas *et al.*, 2016) with MAFFT 7.307 (Katoh and Standley, 2013), respectively. *De novo* maximum likelihood trees from an concatenated AprBA alignment were calculated with FastTree 2.1.9 using the LG model and 1000 resamplings (Price *et al.*, 2010). Only the cluster containing described sulfate-, sulfite- or thiosulfate-reducing *Firmicutes* and *Deltaproteobacteria* species is shown (Watanabe *et al.*, 2016). Branch supports equal to or greater than 0.9 are indicated by black circles. Locus tag and UniProt accession number for each sequence are shown.

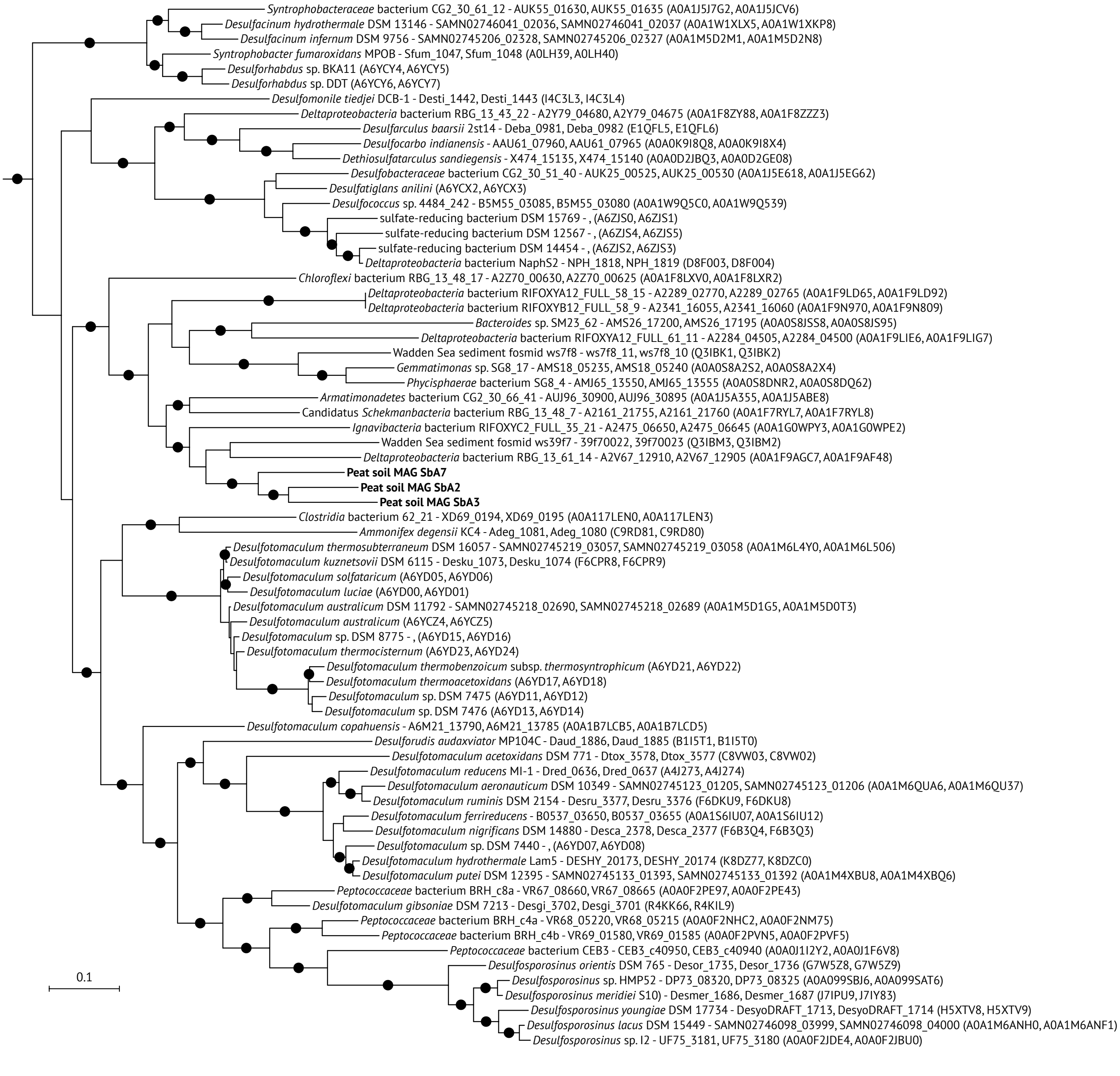

Supplementary Figure S5

DsrD phylogeny and sequence conservation. Maximum likelihood tree was calculated using a M-Coffee alignment (Moretti *et al.*, 2007) and IQ-TREE (Nguyen *et al.*, 2015) with ultrafast bootstrapping (n=1000). Branch supports equal to or greater than 0.7 are indicated by black circles. Locus tags and UniProt accession numbers for each sequence are shown. A subset of the alignment (*Desulfovibrio vulgaris* Hildenborough DsrD, locus tag DVU_0404, amino acids position 5–70) containing the hydrophobic core is shown. Positions of conserved, hydrophobic residues are marked with asterisks (Mizuno *et al.*, 2003).

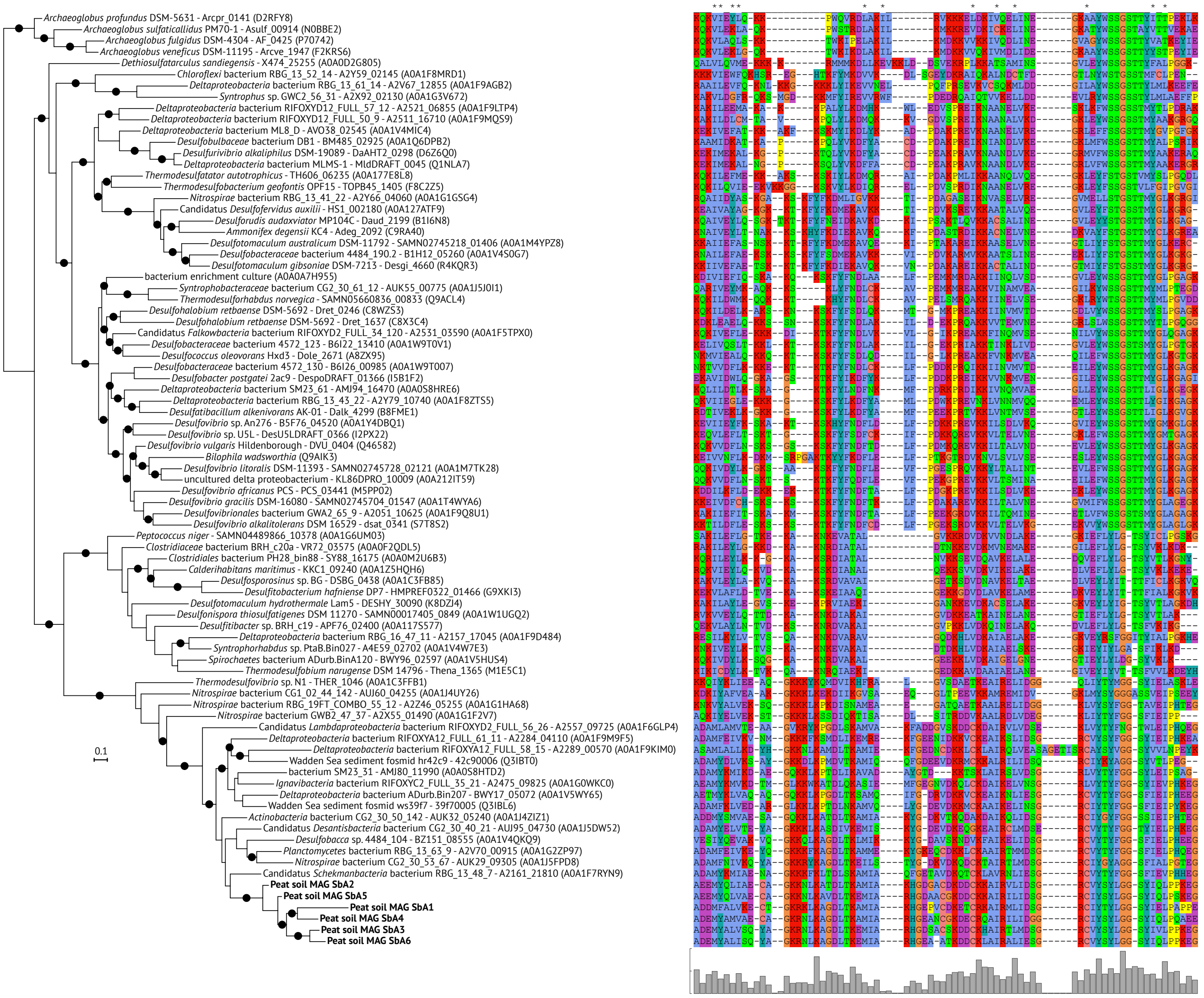

Supplementary Figure S6

DsrL phylogeny and sequence conservation. *De novo* maximum likelihood trees were calculated with FastTree 2.1.9 using the LG model and 1000 resamplings (Price *et al.*, 2010). Branch supports equal to or greater than 0.9 are indicated by black circles. Locus tags and UniProt accession numbers for each sequence are shown. Sequences were reference-aligned to EggNOG's trimmed COG0493 alignment (Huerta-Cepas *et al.*, 2016) with MAFFT 7.307 (Katoh and Standley, 2013), used for phylogenetic calculation, and the subset containing the DsrL of A. *vinosum* and the MAGs was extracted. A M-Coffee alignment (Moretti *et al.*, 2007) of this subset is shown. The N- and C-terminal ferredoxin domains as found in *Allochromatium vinosum* are indicated as blue bars below the alignment (IPR009051 and IPR017896, respectively).

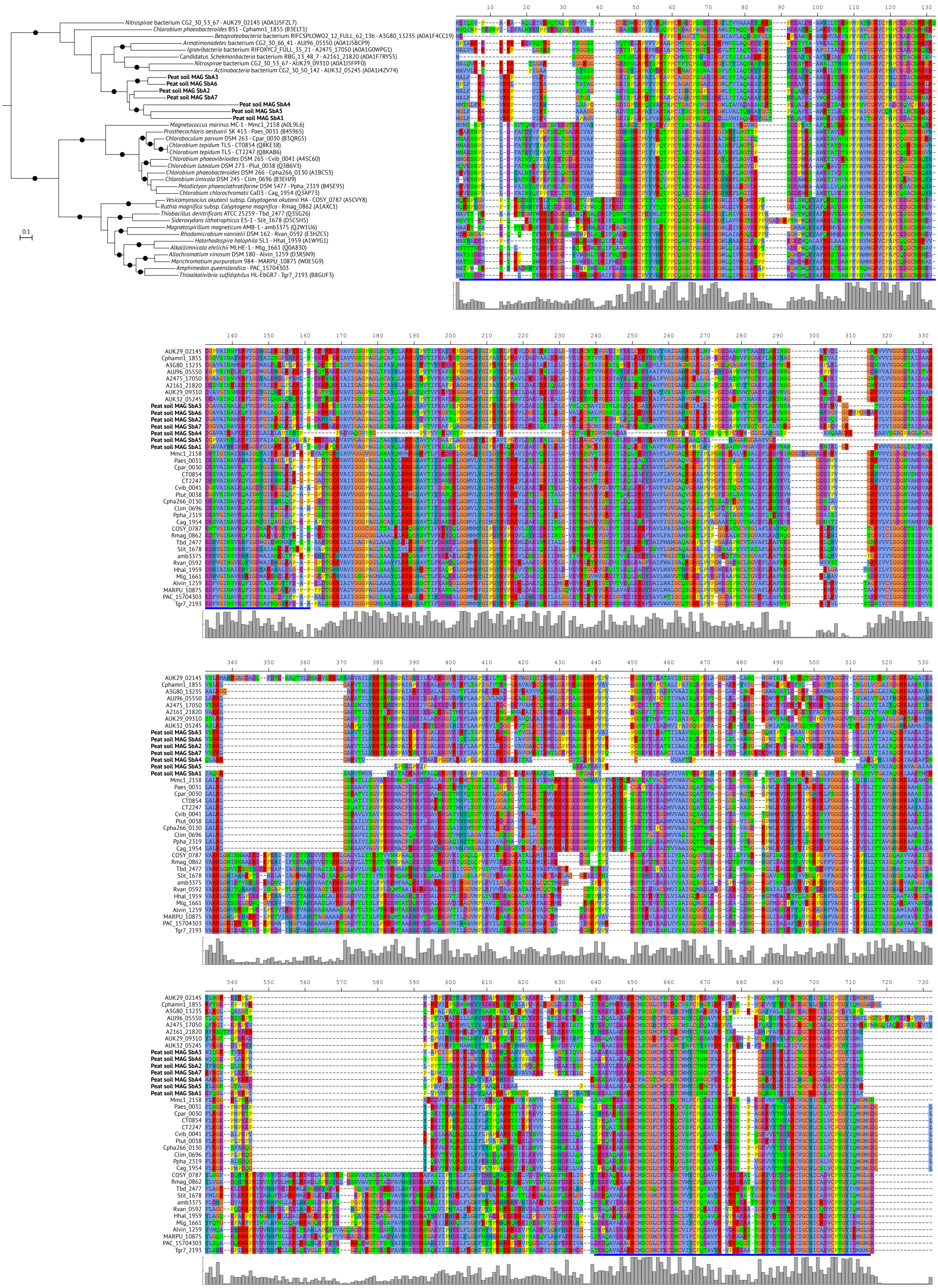
Nitrospirae bacterium CG2_30_53_67 - AUK29_02145 (A0A1J5FZL7)
Chlorobium phaeobacteroides BS1 - Cphamn1_1855 (B3ELT1)
Betaproteobacteria bacterium RIFCSPLOWO2_12_FULL_62_13b - A3G80_13235 (A0A1F4CC19)
Armatimonadetes bacterium CG2_30_66_41 - AUJ96_05550 (A0A1J5BCP9)
Ignavibacteria bacterium RIFOXYC2_FULL_35_21 - A2475_17050 (A0A1G0WPG1)
Candidatus Schekmanbacteria bacterium RBG_13_48_7 - A2161_21820 (A0A1F7RYS5)
Nitrospirae bacterium CG2_30_53_67 - AUK29_09310 (A0A1J5FPF0)
Actinobacteria bacterium CG2_30_50_142 - AUK32_05245 (A0A1J4ZV74)
Peat soil MAG SbA3
Peat soil MAG SbA6
Peat soil MAG SbA2
Peat soil MAG SbA7
Peat soil MAG SbA4
Peat soil MAG SbA5
Peat soil MAG SbA1
Magnetococcus marinus MC-1 - Mmc1_2158 (A0L9L6)
Prosthecochloris aestuarii SK 413 - Paes_0031 (B4S965)
Chlorobaculum parvum DSM 263 - Cpar_0030 (B3QRG5)
Chlorobium tepidum TLS - CT0854 (Q8KE38)
Chlorobium tepidum TLS - CT2247 (Q8KAB6)
Chlorobium phaeovibrioides DSM 265 - Cvib_0041 (A4SC60)
Chlorobium luteolum DSM 273 - Plut_0038 (Q3B6V3)
Chlorobium phaeobacteroides DSM 266 - Cpha266_0130 (A1BCS3)
Chlorobium limicola DSM 245 - Clim_0696 (B3EHJ9)
Pelodictyon phaeoclathratiforme DSM 5477 - Ppha_2319 (B4SE95)
Chlorobium chlorochromatii CaD3 - Cag_1954 (Q3AP73)
Vesicomyosocius okutanii subsp. Calyptogena okutanii HA - COSY_0787 (A5CVY8)
Ruthia magnifica subsp. Calyptogena magnifica - Rmag_0862 (A1AXC1)
Thiobacillus denitrificans ATCC 25259 - Tbd_2477 (Q3SG26)
Sideroxydans lithotrophicus ES-1 - Slit_1678 (D5CSH5)
Magnetospirillum magneticum AMB-1 - amb3375 (Q2W1U6)
Rhodomicrobium vannielii DSM 162 - Rvan_0592 (E3HZC5)
Halorhodospira halophila SL1 - Hhal_1959 (A1WYG1)
Alkalilimnicola ehrlichii MLHE-1 - Mlg_1661 (Q0A830)
Allochromatium vinosum DSM 180 - Alvin_1259 (D3RSN9)
Marichromatium purpuratum 984 - MARPU_10875 (W0E5G9)
Amphimedon queenslandica - PAC_15704303
Thioalkalivibrio sulfidiphilus HL-EbGR7 - Tgr7_2193 (B8GUF3)
0.1

Supplementary Figure S7

Beeswarm plots of expression change of dissimilatory sulfur metabolism genes in anoxic peat soil microcosms. Fold-changes are calculated by pairwise comparisons between replicate metatranscriptomes of the native soil and of each incubation regime and time point. Significant ($p<0.05$) changes are highlighted by back circles.

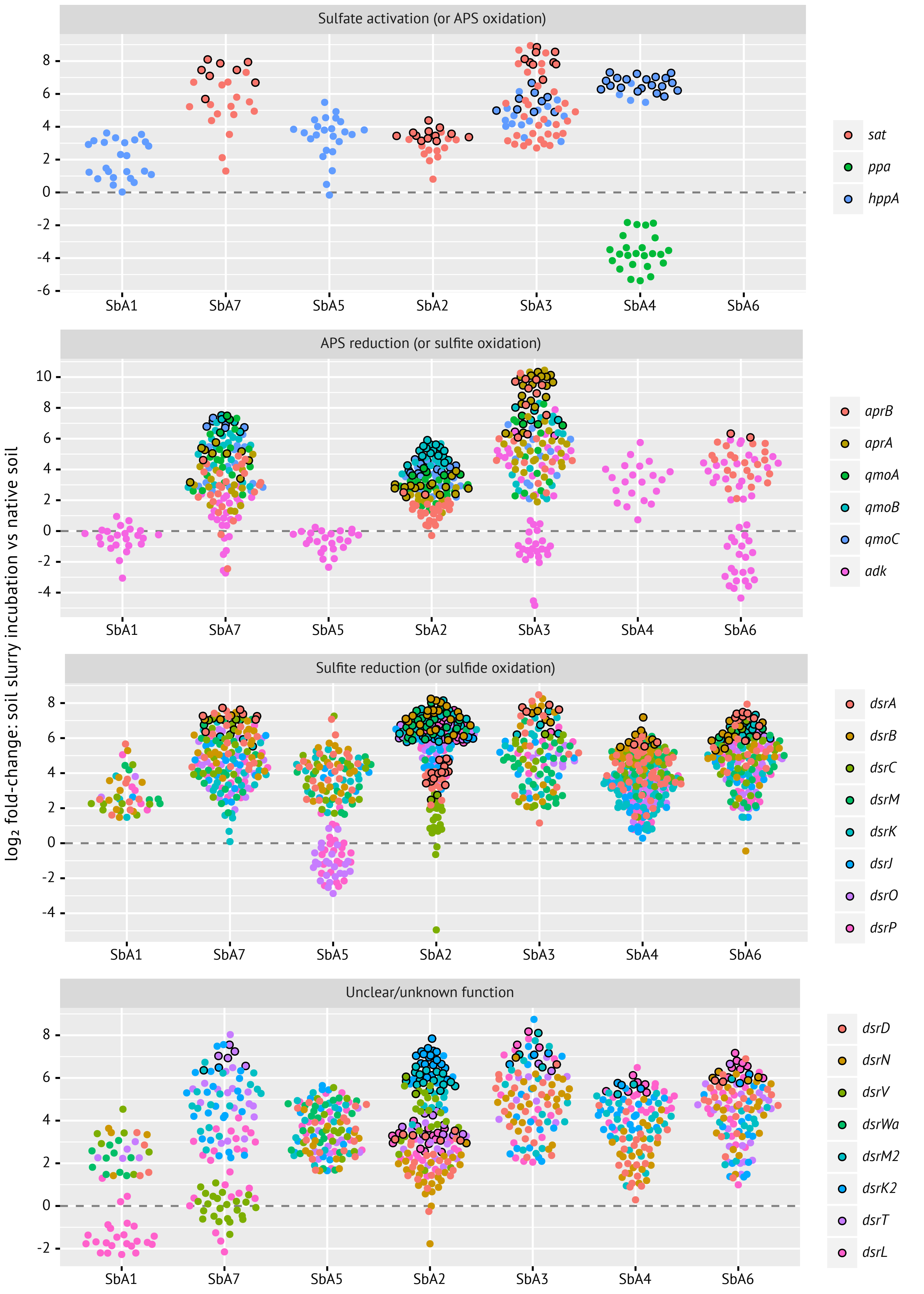
Sulfate activation (or APS oxidation)
sat
ppa
hppA
APS reduction (or sulfite oxidation)
aprB
aprA
qmoA
qmoB
qmoC
adk
Sulfite reduction (or sulfide oxidation)
dsrA
dsrB
dsrC
dsrM
dsrK
dsrJ
dsrO
dsrP
Unclear/unknown function
dsrD
dsrN
dsrV
dsrWa
dsrM2
dsrK2
dsrT
dsrL
SbA1
SbA7
SbA5
SbA2
SbA3
SbA4
SbA6
log₂ fold-change: soil slurry incubation vs native soil
